# Microfluidics for studying metastatic patterns of lung cancer

**DOI:** 10.1186/s12951-019-0492-0

**Published:** 2019-05-27

**Authors:** Monika Ruzycka, Mihaela R. Cimpan, Ivan Rios-Mondragon, Ireneusz P. Grudzinski

**Affiliations:** 1 Department of Applied Toxicology, Faculty of Pharmacy, Medical University of Warsaw, 1 Banacha Street, 02-097 Warsaw, Poland; 2 Biomaterials - Department for Clinical Dentistry, University of Bergen, Årstadveien 19, 5009 Bergen, Norway

**Keywords:** Microfluidics, Lung cancer, Metastasis, Nanomedicine, Nanosafety, Theranostics

## Abstract

The incidence of lung cancer continues to rise worldwide. Because the aggressive metastasis of lung cancer cells is the major drawback of successful therapies, the crucial challenge of modern nanomedicine is to develop diagnostic tools to map the molecular mechanisms of metastasis in lung cancer patients. In recent years, microfluidic platforms have been given much attention as tools for novel point-of-care diagnostic, an important aspect being the reconstruction of the body organs and tissues mimicking the in vivo conditions in one simple microdevice. Herein, we present the first comprehensive overview of the microfluidic systems used as innovative tools in the studies of lung cancer metastasis including single cancer cell analysis, endothelial transmigration, distant niches migration and finally neoangiogenesis. The application of the microfluidic systems to study the intercellular crosstalk between lung cancer cells and surrounding tumor microenvironment and the connection with multiple molecular signals coming from the external cellular matrix are discussed. We also focus on recent breakthrough technologies regarding lab-on-chip devices that serve as tools for detecting circulating lung cancer cells. The superiority of microfluidic systems over traditional in vitro cell-based assays with regard to modern nanosafety studies and new cancer drug design and discovery is also addressed. Finally, the current progress and future challenges regarding printable and paper-based microfluidic devices for personalized nanomedicine are summarized.

**Electronic supplementary material:**

The online version of this article (10.1186/s12951-019-0492-0) contains supplementary material, which is available to authorized users.

## Introduction

Microfluidic and nanofluidic are terms that refer to engineered manipulation of fluid flow that is geometrically constrained to micro-[1] and nanosized objects [2]. These microfluidic and nanofluidic systems are miniaturized devices that are becoming mainstream tools having the potential to recapitulate complex biological processes in vitro and thus influence the improvement of cancer diagnostic and basic cancer research [3–5]. Microfluidic systems have revolutionized three-dimensional (3D) culture techniques [6] and gained greater popularity over traditional two-dimensional (2D) cell culture approaches due to greater ability to reproduce in vivo environment present inside the human body. 3D microfluidic devices in comparison to 2D systems enable fluid manipulation [7], maintaining a controllable temperature [8] and conditions of fresh medium supply [9], shear flow pressure [8, 10] and chemical gradients [6, 11] essential for mimicking processes and mechanism taking place in vivo. The materials that they are made of provide different grades of stiffness and may be permeable to oxygen affecting cell adhesion, migration, and proliferation [6]. Moreover, microfluidic devices can integrate multiple processes such as cell culture handling [6, 12], cell behaviour tracking [13] by real time monitoring [14], simultaneous analysis of several studied groups [9] as well as cell capture [15], lysis [16], detachment [6, 12], mixing [17], and detection [6, 12, 18]. Special properties of microfluidic devices are the possibility of self-organisation in multilayer cellular structures [19] and allowing signal transduction between cells, with the extracellular matrix (ECM) and other systemic factors [12] imitating the structure and physiology naturally occurring in the organism.

**Fig. 1:**
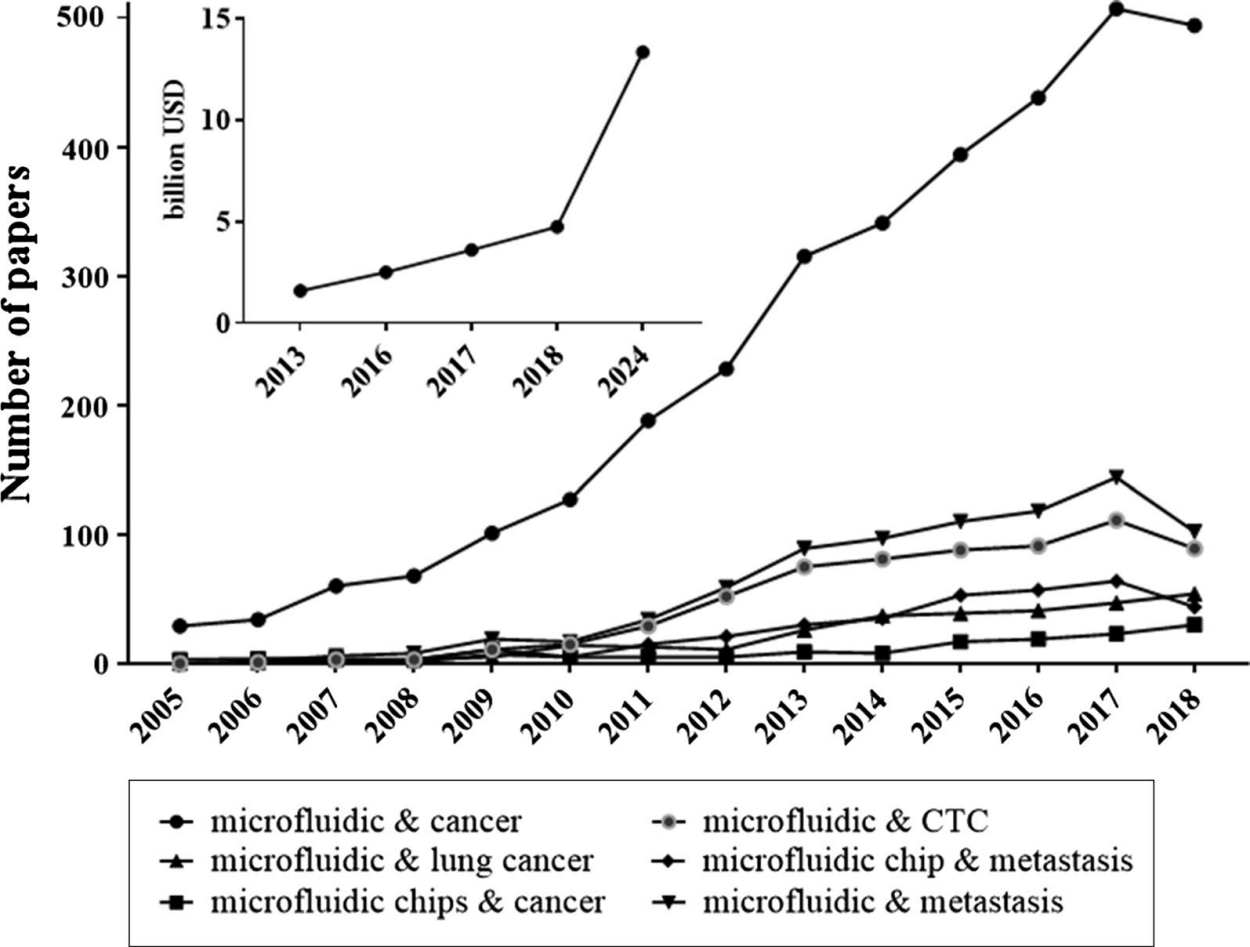
Increased publication trend on microfluidics used in cancer studies in the years 2005–2018. Data were collected based on PUBMED and NCBI databases. The insert presents the estimated and expected worth of the microfluidic market in billion USD based on PR Newswire [33], Grand View Research [20], Markets and Markets [22] and Mordor Intelligence [34] estimations

The beginning of the exploration of microfluidic devices is traced to early 90 s and is currently rapidly progressing. According to Grand View Research [20] and Markets and Markets [21] the worldwide microfluidics market size was estimated at approximately USD 2.5 billion in 2016 and USD 10.06 billion in 2018, respectively. Both, Grand View Research and Markets and Markets expect that the market will expand at a Compound Annual Growth Rate (CAGR) of around 18.4% before 2024 [20] and 22.6% before 2023 [22], respectively, reaching USD 27.91 billion by 2023 [22]. The microfluidic devices are getting more and more popular compared to traditional size equipment due to lower power and time consumption, wider flexibility [23], minimized sample and reagents consumption [4], reduced manufacturing and handling costs [24] along with keeping the features of rapid sample processing [4], automation [24] high throughput screening, high resolution and high accuracy [8] as in the traditional ones. The potential of microfluidic devices has also been noticed by well-known companies like Abbott [20], Merck [25], Roche Diagnostics [20], Ibidi [26], Cepheid, Becton and Dickinson and Company (BD) [20], which provide microfluidic devices. There are also companies developed quite recently, specialized in nanotechnology and microfluidics production such as Darwin Microfluidics [27], Micronit [28], uFluidix [29], Elvesys [30], Micralyne [31] and Dolomite [32]. The increasing demand of microfluidic systems is also reflected in the growing amount of papers concerning engineering of new microdevices and their applications (Fig. 1).

The microfluidic platforms were able to evolve as a result of novel micro/nanofabrication technologies based on different soft lithography techniques [35, 36]. These techniques allow to manufacture the physical objects with dimensions measured in micro- and nanometer scales [37]. Most of the microfluidic devices comprise of chambers [38], channels [39] and other structures such as pillars [40], rods, tubes and wires fabricated as small nanoscale objects [41, 42]. The application of these nanoscale elements in one microfluidic device enables to observe the cancer cells behavior in response to a variety of factors and stimuli in real-time.

The development of microfluidic techniques is predominantly noticeable in relation to micro-engineering devices used for the separation of circulating tumor cells (CTCs). Until recently, most of the microfluidic tests for CTCs enrichment and enumeration were based on the immune-affinity and size-dependent methods commonly used for cancer cell separation [42]. The breakthrough in engineering of the microfluidic systems is the possibility of impedance measurement of each captured cancer cell. Because the dielectric signal obtained from each examined cell has some specific properties, such an approach permits better distinguishing the cancer cell from other type of cells including blood cells [43].

**Fig. 2:**
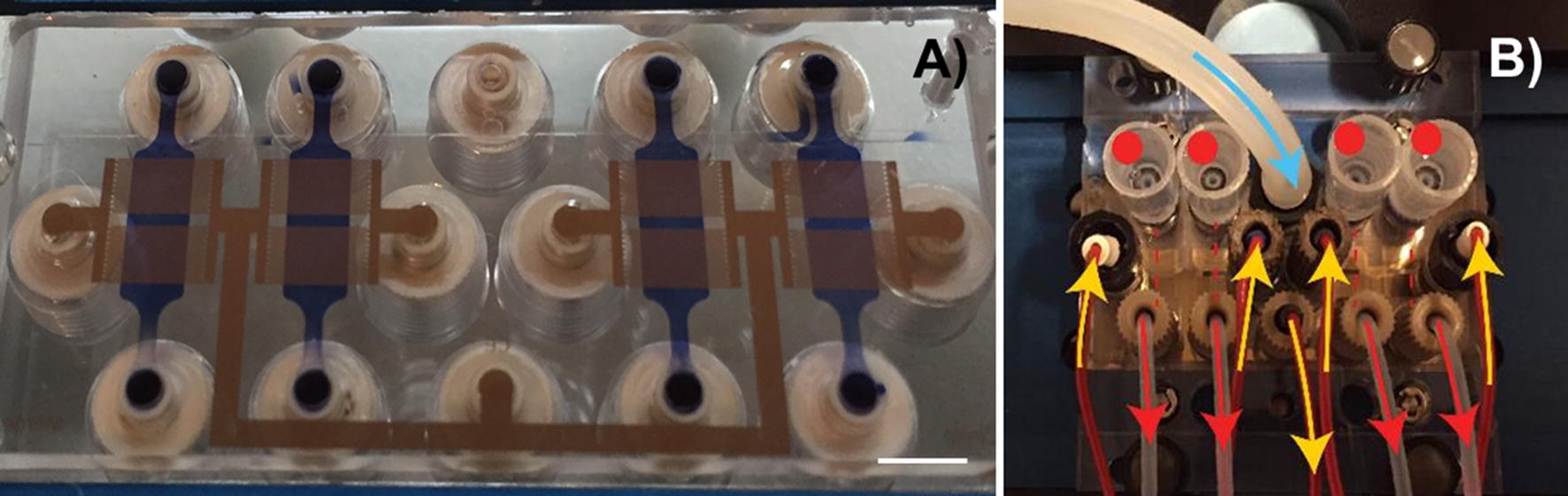
Microfluidic device for nanotoxicity testing originally designed in the GEMNS project (EuroNanoMed II program) by the Nanotoxicology group at the University of Bergen, Norway. Microfluidic set-up. **A** The microfluidic chip comprises four independent microfluidic channels (blue). Cells growing within the microfluidic channel are analyzed via cell-substrate electrical impedance using microelectrode arrays (gold) (scale bar, 5 mm). **B** Mounted microfluidic chip. On-chip liquid reservoirs (red dots), tubing from syringe pumps (red arrows), electrical contacts (yellow arrows) and tubing providing humidified air/CO_2_ are connected to the chip

More sophisticated and advanced microfluidic-based platforms are continuously developed. The latest chips are even capable of biomimicking some biological processes, such as metastasis [44] and recapitulating the physiological activities of entire organs [45] and of the human body [46]. This revolutionary approach in point-of-care (POC) diagnostics allows a detailed study of simple mechanisms at the cellular level, as well as of complex processes involved in diverse tissues and organs. Moreover, microfluidic systems may also serve as a platform for drug development and nanosafety assays (Fig. 2) [45, 46]. The development of microfluidic techniques may contribute to the reduction of animal models’ use in cancer research, and more importantly overcome the interspecies limitations in new anticancer drug investigations.

Lung cancer is a leading cancer worldwide [47]. According to the American Cancer Society, at least 85% of the total cases of lung cancers refer to non-small cell lung cancer (NSCLC), 10–15% to small cell lung cancers (SCLC), and approximately 2% to lung carcinoid tumors [48–50]. Studies evidence that lung cancer often metastasizes to distant parts of the lung and other organs like bones, brain, liver [51], breast [52], colon [53], kidney [54], and many others [44]. The American Lung Association informs the public that the survival rate in patients with metastatic lung cancer is lower comparing to other leading cancers. The 5-years survival rate for lung cancer without metastasis accounts for 55% [55], while for metastatic lung cancer accounts for approximately 4% [56]. The aggressive progression of lung cancers [47], resistance to chemotherapy [57] and high mortality ratio in cancer patients has recently drawn attention of scientists to thoroughly investigate and identify the mechanism of lung cancer development in order to develop targeted therapies.

Here we present the first comprehensive overview of microfluidic systems and their applications for studying the complex biological mechanisms that occur during lung cancer metastasis. The review also outlines recent scientific achievements regarding metastatic processes and advancements in anticancer drug development. It also presents nanosafety issues in modern nanomedicine.

## The metastatic process of tumor cancers

Metastasis is a sequence of cellular and molecular events leading to cancer outgrowth in distant sites and organs of the body. This process is launched from the dissemination of primary tumor cells that undergo transformation, acquiring aggressive traits like ability to move and penetrate into the extracellular matrix, resulting in the development of secondary tumors [58, 59]. Briefly, it is a complex and multi-stage process that is initiated by the cancer cells’ breakaway from the tumor tissue (epithelial–mesenchymal transition), subsequent cancer cells intravasation, survival within the bloodstream and cells’ migration toward various sites in the organism, extravasation and development of metastatic foci [58, 60–62]. This aggressive and invasive process is the final consequence of a bi-directional communication between the cancer cells and the surrounding tumor microenvironment that is manifested by a tumor microenvironment evolution responding to disease initiation and progression towards the invasion stages [60].

The initiation of the metastasis process is conditioned by many factors including gene expression and cellular components. Cells with metastatic potential feature high heterogeneity due to genetic alteration involving chromosomal reorganizations, DNA mutations and epigenetic modifications. Such genes undergo expression at the late state of cancer development, which can be explained by gaining invasive traits for seeding at distant sites [63]. The extreme importance in metastasis initiation and progression is the intercellular crosstalk between cancer cells and the surrounding tumor microenvironment. In the first stages of metastasis, disruption of cell–cell adhesion mediates uncontrolled cell growth promotion and cancer cells’ dissemination. In this catenin-dependent process cells undergo epithelial–mesenchymal transformation gaining migratory abilities [64]. Once the cancer cells obtain the ability to move away they invade the adjacent extracellular matrix (ECM) and intravasate into blood stream and lymphatic system. This process is supported by cytokines, growth factors, matrix metalloproteinases (MMPs), integrins, actin binding proteins and chemokines [62, 63] frequently mediated by tumor-shed exosomes [65]. The tumor-associated stroma cells such as macrophages, fibroblasts, vascular space-related cells, and various immune responses are also involved as they are governed by the state of tumor progression [63, 66]. The stromal cells further enhance the aggressive behavior of carcinoma cells through various heterotypic signaling such as IL-6, CD4^+^ and IL-4 [59].

Reaching the circulatory system by tumor-derived cells through the intravasation process is mostly associated with their quick destruction, among others due to exposition to shear stress in the vasculature. Therefore, less than 0.1% of circulating tumor cells are capable to form secondary tumors. Probably, the ability to survive belongs to those CTCs that underwent epithelial–mesenchymal transition (EMT) and evaded the anoikis processes [67, 68]. The CTCs mostly spread out through blood or lymphatic vessels, however there are also other ways such as transcoelomic dissemination into the pleural, pericardial, and abdominal cavities [67]. In a place where the blood flow is reduced, CTCs adhere to vascular endothelium, express adhesion molecules and lunch the production of holes in endothelia for escaping from vasculature towards the preferred organ [68]. The selection of the susceptible metastatic organ was revealed to be associated with tumor-induced determination of the microenvironment, named pre-metastatic niches (PMNs). The PMNs initiation and organization prior to CTCs seeding involves various environmental components, signaling factors and tumor-secreted vesicles [69]. In the case of disseminated lung cancer cells, their main places of destination are thought to be the brain, bones, adrenal glands and liver [70].

**Fig. 3:**
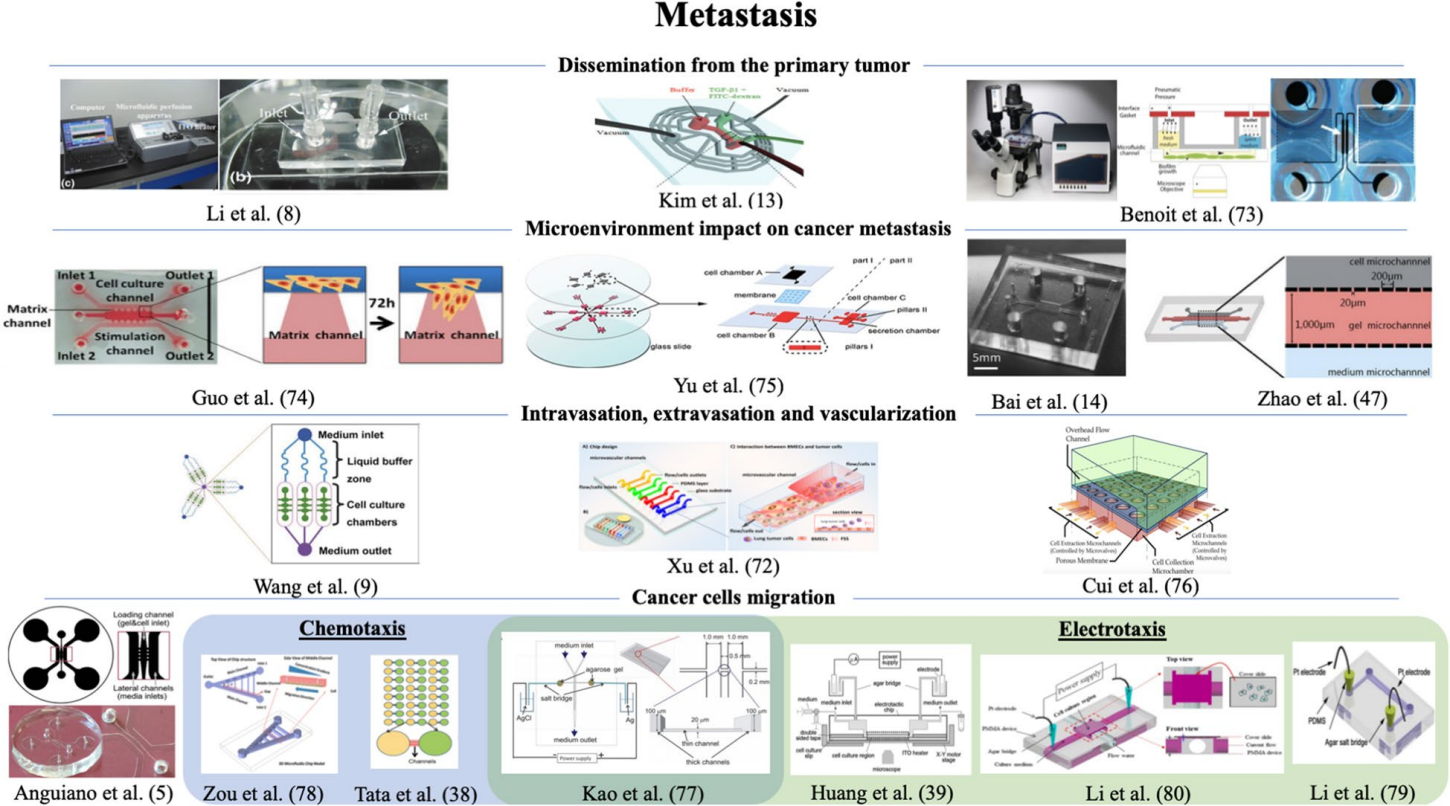
Graphical representation of the microfluidic devices used in cancer studies described in the chapter 2. Presented microfluidic systems were applied to study the different stages of lung cancers metastasis. The figures are reproduced from Li et al. [8] with permission of Applied Biochemistry and Biotechnology, Kim et al. [13] and Xu et al. [72] with permission of Electrophoresis, Benoit et al. [73] with permission of Applied and Environmental Microbiology, Guo et al. [74] with permission of Biochemical and Biophysical Research Communications, Yu et al. [75] and Bai et al. [14] with permission of Oncotarget, Zhao et al. [47] with permission of Scientific Reports, Wang et al. [9] and Anguiano et al. [5] with permission of Plos One, Cui et al. [76] and Kao et al. [77] with permission of Biomicrofluidics, Zou et al. [78] with the permission of Analytical Chemistry, Tata et al. [38] with permission of Advances in Natural Sciences: Nanoscience and Nanotechnology, Huang et al. [39] and Li et al. [79] with permission of Biosensors and Bioelectronics, Li et al. [80] with the permission of Analytical and Bioanalytical Chemistry

Once the cancer cells undergo rapid growth in the invaded tissue the colonization is already initiated in the distant organ. Angiogenesis and lymphangiogenesis have a pivotal meaning in this step. New vasculature formation is started by local injuries in the basement membrane by rapid destruction and hypoxia processes. Subsequently, endothelial cells migrate and proliferate, mediated by angiogenic factors, since tumor requires constant nutrition and oxygen supply [71]. The microfluidic devices applied to study the different stages of lung cancers metastasis described in this overview are presented in Fig. 3.

### Dissemination from the primary tumor

The epithelial–mesenchymal transition is the key point for cancer cells moving out from the primary tumor. During this process the cells acquire migratory abilities that make them capable of invading local and distant tissues [60]. Losing of cell polarity, rearrangement of cell–cell junctions, cells elongation and gaining of a fibroblast-like morphology are elements of a multistep EMT process that is modulated by extracellular signaling factors including receptor tyrosine kinase (RTK), transforming growth factor β (TGFβ), and Notch and Wnt signaling pathways [62, 81]. These molecules can co-operate inducing expression of transcriptional factors that subsequently lead to the down regulation of the epithelial phenotype (E-cadherin) and upregulation of the mesenchymal phenotype. The appearance of the mesenchymal phenotype is expressed by production of N-cadherin and vimentin. This in turns supports tumor growth, the rearrangement of cytoskeletal organization and supports the promotion of metastasis [62].

The TGFβ participations in EMT activation was reported in many tissues and is being considered the most prevalent mediator of the EMT process. Therefore, the regulatory mechanisms of TGFβ need deeper investigation, which is why Kim et al. developed a microfluidic gradient device to reproduce the association between TGFβ and EMT [13]. The device involved a vacuum channel network and fluidic microchannels for the generation of a stable concentration gradient. The application of the device enabled to establish the TGFβ concentrations that initiated and fully converted cells to mesenchymal form. The influence of the various concentrations on the EMT process was analyzed by cells’ elongation monitoring, as well as the expression of epithelial and mesenchymal factors by Western blot.

A special importance of the EMT process occurrence is assigned to the strength of cell adhesion. Recently, Li et al. [8] constructed a microfluidic chip for the measurement the adhesion force of cells. The device consisted of a microfluidic system coupled to a temperature controllable perfusion apparatus and parameter interface. The mechanism of operation was based on the creation of controllable fluid shear force conditions within the microchannel. This device enabled to investigate the adhesion capacity of A549 cells (adenocarcinomic human alveolar basal epithelial cells) on proteins being components of extracellular matrix, such as laminin, collagen IV, ECM Matrigel, fibronectin, and 2% BSA. The adherent forces of the studied cells were weakest in BSA. The strength of adherence was rising in fibronectin, ECM Matrigel and collagen IV, respectively. The strongest binding was observed for laminin as well together with a concentration-dependent increase of shear stress. The following step of investigation revealed a TGF-β interruption in the interaction between A549 cells and laminin in a time-dependent fashion. In order to confirm the obtained results, the labeling of filamentous actin from cytoskeleton and vinculin, i.e., the focal adhesion protein, during the incubation of epithelial A549 cells with TGF-β was performed. The loss of cell-extracellular protein adhesion was observed proving the launching of EMT process. Summing up, the device was successfully applied, and more than that, the constructors claimed that the device also possessed the ability to investigate the adhesion between different kinds of cells.

The mechanism of EMT was also studied by Breiman et al. who investigated the participation of fucosylated antigen expression on the epithelial and mesenchymal state in cancer evolution [10]. The researchers revealed the relation between surface neutral α1, 2 and α1, 3/4 fucosylated glycans and epithelial state of MCF10A cells with conventional methods (immunofluorescence, flow cytometry, qRT-PCR). They suspected that this interaction can be mediated by endogenous lectins, such as prolactin (lectin CLEC17A), hence they investigated this issue using the BioFlux microfluidic device. The BioFlux device was created for simultaneous biofilm growth with a controlled shear flow pressure and cells viability [73].

### Microenvironment impact on cancer metastasis

The tumor microenvironment has a great impact on metastasis initiation, tumor cells proliferation, and their further migration and colonization of distant tissues [60]. An absolutely fundamental meaning has the recruitment of microenvironment components, such as tumor-associated macrophages (TAMs) and cancer-associated fibroblasts (CAFs). Those and many others stromal components, such as immune suppressor cells and chemokines stimulate the metastatic process [62, 82].

Tumor-associated macrophages have been reported to play a special role in tumor cell invasion. They derive from normal macrophages (M1) that possess the anti-tumorigenic potential. Interestingly, under unknown circumstances they are capable of changing their profession and acquire a tumor-promoting form (M2). The M2 macrophages are further subcategorized into M2a, M2b and M2c, based on the various factors that are responsible for the promotion of their polarization [14]. The M2 mechanism of action in metastasis was found to be associated with the facilitation of tumor cells’ invasion via paracrine signaling. This signaling involves CSF-1, EGF and proteases, such as cysteine and cathepsins that enhance tumor progression [82]. However, the issues of macrophages’ phenotype switching and their specific role in carcinoma cell dissemination remain unknown. Recently, Bai et al. constructed a three-dimensional (3D) microfluidic platform to investigate the relation between distinct TAMs in inducing EMT and cancer cells spreading [14]. The 3D microfluidic system incorporated a dynamic camera for real-time monitoring of the interactions between carcinoma cells and macrophages, visualization of cancer cell aggregate distribution and precise measurement of cell–cell distances. The device consisted of 2 inner and 2 outer channels. It was built in a way to allow the “contact condition” and “separated condition” circumstances enabling macrophages to be positioned in a direct contact or separated from carcinoma cells aggregates. In details, the inner channels were filled with type I collagen gel solution in order to create a “contact condition”. One of the outer channels was devoted for HUVECs culturing and growing in EGM-2 endothelial cell growth media. The tumor cells and macrophages were subsequently introduced into the inner channel that was the farthest from the previously described outer channel. Last outer channel was filled with DMEM that was changed on a 24-h cycle. In order to create a “separated condition” the macrophages and tumor cells were introduced separately into different inner channels. Using this device, Bai et al. also demonstrated the distinct roles of macrophage subtypes in the induction of the EMT phenotype of lung carcinoma cells. M1 and M2b macrophages were highly capable of inducing EMT, regardless of the studied conditions. Interestingly, M2a was established to possess the fastest and preferential migrated abilities toward carcinoma aggregates as well as to promote cells aggregate dispersion in greater majority in “contact” conditions. The greatest achievement of their study was to evidence the M2a promoting impact on carcinoma cells dissociation through a CD11b and ICAM-1 interaction.

Fibroblasts are the components of connective tissue, however in cancer modified stroma they acquire an aggressive phenotype, named cancer-associated fibroblasts (CAFs), which demonstrate mesenchymal-like phenotypes and are capable of promotion of tumorigenesis [82]. To address this issue, Yu et al. engineered a 3D co-culture microfluidic system to emulate an in vitro tumor microenvironment [75]. The proposed device enables to investigate the interactions of cancer cells and various cellular components of stroma in real time. Their work aimed to elucidate the influence of CAFs on human lung adenocarcinoma progression. The ability of the device to create the in vivo microenvironment was examined by normal human fibroblasts activation to CAFs and measurement of myofibroblast markers concentration. In the subsequent steps the device provided the information about enhanced NSCLS cells motility, promoted by CAFs-secreted components. The obtained results conjectured that CAFs influence the progression in human lung carcinoma cells via increased expression of GRP78.

A significant role in tumor progression is played by transmembrane receptors. CD47 is the ligand for the receptors expressed on the macrophages and other phagocytic-like cells that inhibits macrophage phagocytosis causing the cancer metastasis propagation [47, 83]. The overexpression of CD47 was reported in various malignances and its contribution in the progression of NSCLC has recently been investigated by Zhao and collaborators [47]. They fabricated a microfluidic chip that served for the measurement of migration and invasion of intact NSCLC cells, siRNA NSCLC and NSCLC cells with overexpression of CD47. The chip involved three microchannels and two rows with micro-gaps that permitted cells extravasation following the chemical gradient. The first utmost chamber served for cells seeding, the inner chamber was filled with Matrigel and the last contained the chemoattractant FBS. Their results revealed that siRNA down-regulated the CD47 expression leading to suppression of cells’ migration/invasion, while overexpression of CD47 significantly enhanced cells’ migration and invasion abilities. Moreover, in vitro the results obtained in the chip and the in vivo obtained from on a mice model were consistent and indicated a metastasis inhibition by targeting CD47.

Still, many regulatory factors that promote metastasis on a molecular level have remained undiscovered. Recently, Guo et al. investigated the impact of the transcription/translation factor Y-box-binding protein-1 (YBX1) on NSCLC aggressiveness [74]. One of the stages of the experiment was the development of a microfluidic model for studying the YBX1 mediated lung cancer cells invasion. The aggressiveness of stimulated cells was indicated by an increase in the invasive distance and area. The authenticity of the obtained results was proven in in vivo studies.

### Transendothelial migration and vascularization

#### Intravasation and extravasation

Intra- and extravasation are the processes of cells transmigration into vasculature and out of vasculature, respectively. The first one gives rise to cells spreading through the vessels, and the second results in seeding the distinct localization by primary tumor cells. The movement of tumor cells through endothelium is supported by the participation of neutrophils and CB11b^+^. Neutrophils produce the matrix metalloproteinase-9 (MMP-9) [84], while dendritic cells CB11b^+^ participate in directing of cancer cells toward distant sites in the organism. Moreover, the tumor cells’ closeness to the endothelial barrier [85] and expression of cytokines such as TGFβ augment the vessels permeability [62]. Cancer cells are also capable of secreting protein angiopoietin-like-4 (Angptl4), EREG, COX-2, MMP-1, and MMP-2 that may facilitate extravasation. Pulmonary hyper-permeability induction is associated with angiopoietin2 (Angpt2), MMP-3, MMP-10, placental growth factor, VEGF, and inflammatory monocytes [59].

A significant meaning during cells intravasation and extravasation has the formation of invadopodia, that are able to penetrate the basement membrane via localization of proteases and extracellular matrix degradation [9, 86]. There are many factors involved in invadopodia formation (e.g., EGF, matrix metalloproteases, PDGF, PKC, N-WASP, ERK), however the molecular mechanism of their formation remains unclear [9]. Wang et al. engineered a microfluidic device for 3D culture to study the process of invadopodia formation by the human non-small cell lung cancer cell line A549 [9]. Cells culturing in a 3D mode was aimed at mimicking the in vivo microenvironment, thus the device was made from polydimethylsiloxane (PDMS) in order to provide a conducive condition for cell growth and proliferation. The fresh medium was constantly supplied at a control flow rate by an injection pump. An ECM microenvironment was obtained by Basement Membrane Extract (BME) substitution, since it contains ECM-like compounds. The device consisted of three units with a mutual outlet. Each unit possessed its own inlet for compounds application and medium addition. The construction of the device enabled the simultaneous performance of the analysis on three different studied groups: control group, EGF and GM6001/EGF groups. EGF was studied because of its stimulating properties for cell growth and cell motility, while GM6001 was used as a MMP inhibitor. The assessment of invadopodia formation of the cells was performed by detection of F-actin and cortactin expression by subsequent immunofluorescence. The invadopodia morphology was inspected by confocal imaging system. The obtained results provide evidence that EGF induces A549 cancer cell invadopodia formation and can be greatly inhibited by GM6001. The microfluidic system devised by Wang et al. enables the exploration of invadopodia formation to facilitate the understanding of the invasion mechanisms in metastasis and discovery of anti-invasion therapeutic methods.

Brain metastases are one of the common secondary sites caused by lung cancer dissemination [87]. For that reason, the understanding of the interaction between lung tumor cells and the brain microvasculature wall in the process of extravasation could be a critical step in the development of new drugs that could serve as inhibitors of this process. Therefore, Xu et al. [72] replicated the biochemical and mechanical traits of inflammatory brain microvascular endothelial cells (BMECs) in a microdevice to investigate the regulatory mechanisms and possible signaling pathways of the rolling and adhesion behavior of lung tumor A549 cells. The recapitulation of inflammatory BMECs was achieved by stimulation with TNF-α and application of fluidic shear stress. Their results demonstrated that lung tumor cells’ interaction with inflamed endothelium and lesion place is the key point in the extravasation process, however this can be reversed after Rho/ROCK inhibitor administration.

Microfluidic platforms have also been used to study intra- and extravasation in relation to different cancers cells, such as the breast cancer-cell lines: MDA-MB-231 [85, 88], colon cancer-cell line: LOVO and SW480 [89], and salivary gland adenoid cystic carcinoma-cell line: ACC-M [90]. Recent refinements in the microfluidic systems construction for a closer understanding of the mechanisms of intra- and extravasation were performed by Cui et al. [76] and Chen et al. [58]. Cui et al. proposed a microengineered device comprising multiple independent chambers and a biocompatible porous membrane for the selective gathering of the cells that migrate through endothelium [76]. The transendothelial migratory capability of MDA-MB-231 cell line was observed under the circumstances of a defined shear stress and in sub-regions with the full coverage of endothelial layer. The results obtained from the experiment demonstrated differences in body aspect ratio, planar migration, stress fiber alignment, and nuclear paladin expression in comparison to cell with non-migratory phenotype. Interestingly, the device can be configured for other cells examination and has potential for biofluids like serum and whole blood investigation. An innovative approach concerning transendothelial migration was reported by Chen et al. [58]. They developed a microengineered system with the capability of self-organized human microvascular networks creation and an easy differentiation between extravasated, mid-extravasated and intravasation cells using standard confocal microscopy. The construction of the device ensured the quantification of tumor cells kinetic data (protrusion initiation rate and speed of complete transmigration), fast imaging, and accurate extravasation scoring.

#### Neoangiogenesis

Microfluidic systems that imitate vascularisation formation are restricted to a few cases [91] which mostly consider non-cancer-related matters [92–94]. However, neoangiogenesis is a key step in tumor development and metastasis. During this process new capillaries are formed allowing tumor cell growth by nutrients and oxygen delivery [88]. This process is signaling-dependent [62] and involves the incorporation of pre-existing endothelial cells, the recruitment of progenitor cells from endothelium and transendothelial migration toward the metastatic place [95]. The surrounding environment undergoes stimulation by neoangiogenesis factors, however they do not have to be directly generated from tumor cells [62]. Examples of neoangiogenesis stimulators are bFGF, VEGF, TGFβ-1, HGF, TNF-α, PDEGF, angiogenin [96, 97] and Il-8 [96, 98, 99]. These interactions concerning signaling factors releasing, targeted cell migration and reorganization of the environment next to the metastatic place are strongly associated with cell communication. Heterotypic (cells of different type) cell-to-cell interactions are observed between cancer cells as well as with the extracellular matrix during vasculogenesis. Vascularization can also occur in alternative ways by glomeruloid microvessel growth, vasculogenic mimicry, intussusceptive microvascular growth, postnatal vasculogenesis and vessel co-option [100].

The complexity of the vasculogenesis process was investigated by Alonzo et al. who constructed a microfluidic system for this purpose [91]. The results revealed that vessel network formation depends on interstitial flow mediated communication and stromal cells participation. The system enabled to study the interstitial flow influence on heterotypic and homotypic cell–cell interactions on the vasculogenesis. Moreover, the device delivered an initial spatial and temporal pattern for studying the interactions between cells participating in vasculogenesis and the surrounding environment. The device also gives the possibility for microenvironments’ isolation in the 3D model.

### Cancer cells migration

Migration is a critical process of metastasis that proclaims the invasive phenotype of cancer cells [62]. It is a complex process that is extremely sensitive to extracellular matrix and media stimulation [101]. Integrins, ion channels, cell adhesion molecules, soluble cytokines, growth factors, matrix-degrading proteases, and Rho GTPases [62, 79, 102] are the molecules that mediate cancer cells’ migration. Integrins are accountable for the transduction of mechanical signals from ECM into cells via focal adhesions and other macromolecular complexes. The focal adhesions in turn may modulate cell motility by activation of kinases such as FAK and PI3K. PI3K thus may activate Rho that belongs to Rho family GTPases. The Rho activates ROCK that correlates with the promotion of tumor metastasis. Physical features of some focal adhesions such as shape and size may also influence the targeting of cell movement. Polymerization of actin cytoskeleton is another predictor of cell motility. Similarly does cofilin, one of the actin-binding molecules, that is also engaged in cell migration promotion under optimal expression [62].

There are three different types of migration modes: epithelial (non-migratory), mesenchymal (migratory) and amoeboid known also as lobopodial (squeezing motility) [103], however the processes of mode switching have not been well explained. A microfluidic device that allows the investigation of the impact of different microenvironmental conditions on cancer cells migration mode and measure H1299 lung adenocarcinoma cancer cells’ motility by quantitative image analysis was proposed by Anguiano et al. [5]. Their chip has a central chamber serving as an inlet for loading of hydrogels and cells, and lateral channels for serum insertion. Microenvironments were recapitulated by the application of three types of hydrogels. One hydrogel contained pure collagen type I (C), and the next two were obtained by the fusion of collagen type I and Matrigel (CM—1:1/collagen:Matrigel, CM+—1:2/collagen:Matrigel). Matrigel was recommended for 3D culturing. It is a kind of a basement membrane rich in fibrin, collagen and a number of growth factors that may serve as a microenvironment after cancer invasion [5, 104]. Researchers noticed that exposition to relatively more cross-linked environment (CM) resulted in faster cells migration coinciding with a display of the lobopodial phenotype. They explained this phenomenon as an impact of environment rigidity, increased pore size, and appearance of soluble factors that activate the GTPase RhoA pathway. However, a further increase of hydrogel stiffness (CM+) resulted in movement impairment. The changes in type switching from mesenchymal to amoeboid were explained by referring to the effectiveness of migration. The mesenchymal movement is more effective in smaller pore sized hydrogels, while amoeboid in higher pore sized hydrogels, which enhance the cell migration speed. Moreover, they noted that the Anti-β1 and Anti-β3 integrins blockage modulated the transition from mesenchymal to amoeboid [5].

Migration in metastasis is a directed cells’ movement that can be guided by chemical and electrical cues. Chemical cues are generated by tissue-derived chemical factors, named chemoattractants that form a chemical/biochemical concentration gradient [105]. Although, the biochemical gradients have been noticed to significantly influence the chemotactic cells’ response via cellular morphology and migration rate modification, or gene expression and signaling cascades regulation [78], the complexity of mechanisms involved needs deeper exploration. To study this issue, Zou et al. constructed a microfluidic network that generated multiple stable gradients for the examination of the chemotaxis related migratory responses of lung cancer stem cells (LCSC) and differentiated lung cancer stem cells (dLCSC) [78]. This chip permitted the observation of the migratory behavior of both LCSC and dLCSC in Wnt signaling pathway dependent of β-catenin in real time. The application of different gradients induced different migration cell rates and different response of studied subcultures. Interestingly, dLCSC occurred to be more sensitive for gradient stimulation in comparison to LCSC. Moreover, the application of XAV-939 resulted in inhibition of β-catenin signaling, leading to the suppression of chemotactic migration rates.

The influence of the EGF factor on cell movement in a microfluidic system engineered especially for this purpose was investigated by Tata et al. [38]. The basic units were two chambers linked by ten microchannels. One chamber was dedicated for seeding of lung metastasized prostate cancer (PC3-ML) cells while the second for EGF introduction. Microchannels enabled the observation of cancer cell migration toward the attractant in a concentration-dependent manner. The highest response in cell movement occurred after the application of 100 ng ml^−1^ of EGF.

In recent years, increased interest has been directed to the impact of direct current electrical field (dcEF) on tumorigenesis. It has been reported that cell may undergo reorientation and migration in an electric field-induced manner. This phenomenon is called electrotaxis or galvanotaxis [80]. Physiological values of dcEFs in the animal body occur from 50 to 500 mV mm^−1^ and are produced by transepithelial potentials [77, 106]. It has already been proven that the electric field is involved in embryonal development, wound healing, bone regeneration, and tumor metastasis [39, 105]. To address this issue in context of metastasis, Huang et al. developed a microfluidic device for a long-term electrotaxis study of the human lung adenocarcinoma cell lines CL1-0 and CL1-5 under microscope monitoring [39]. The chip integrated a tightly sealed cell culture chamber and a heater with thermostat. The construction of the chip allowed the application of simultaneous multiple electric fields in a single experiment. The experiment revealed differences between studied subcultures of carcinoma lung cells in response to electric fields stimulation. The CL1-5 cells considered as highly metastatic showed strong electrotactic response while no influence was observed on CL1-0 cells. Moreover, based on the CL1-5 cells specific movement after 1 h of stimulation, it was suggested that different signaling pathways may influence cells orientation and migration.

Li et al. constructed a microfluidic device for mimicking the endogenous environment, where they applied dcEF for examination of the electrotactic migration of non-small cell lung cancer cell lines (H460, HCC827, H1299, and H1975) [80]. The experiment consisted of following steps: (i) glass slide coating with fibronectin, (ii) cells introduction for culturing from 48 h up to 60 h, and (iii) placing electrodes into the medium channel and electrical stimulation. After accomplishing the examination, a quantitative real-time PCR and quantitative analysis of cell migration were performed. The experiment enabled the study of different migration behaviors of cancer cells, as well as the observation of changes in dcEF-induced cell morphology and protrusion formation. The application of dcEF resulted in a migration of H460 and H1975 cells to cathode, while H1299 migrated toward anode. HCC827 had weak anodal directionality. Increased motility and cell reorientation were noted in H1299 and HCC827. Based on mRNA expression they revealed that MAPK and PI3K signaling pathways are associated with dcEF stimulation. Moreover, the relationship of dcEF stimulation with Ca^2+^ signaling in the migratory behavior of lung cancers cells was also reported. Obtained results proved once again the existence of intrinsic heterogeneity within the same cancer cell line. In later stages of their work, Li et al. proposed two microfluidic devices. The first was a more complex microfluidic system for long-term cells migration study under electric field, while the second was addressed to the isolation of subpopulations based on different responses to electric field [79]. New devices maintained controllable microenvironmental conditions and allowed cells’ motility observation in real time. The lung cancer cells H1975 used in this study revealed cathodal migration. Furthermore, their movement seemed to be dependent on the EF stimulation and specific genes expression. The motility of studying cell lines under EF stimulation was related to phosphatidylinositol-3,4,5-trisphosphate 3-phosphatase (PTEN) expression, while the absence of EF was associated with EGFR expression. The investigation enabled to establish that upregulation of RhoA was linked to high cells motility. The second chip allowed the isolation of the cellular subpopulations with different electric induced migration abilities.

An interesting approach was presented by Hou et al., who proposed a multi-conditional microengineered system for simultaneous chemotactical and electrotactical cell stimulation for molecular mechanism exploration [106]. This chip integrated several isolated channels with a single chemical circuit and isolated chemical flow. This approach enabled to establish that the ROCK inhibitor influenced specific suppression of the directedness of CL1-5 cells movement. Moreover, the addition of the PI3K inhibitor resulted in the suppression of both the directedness and migration speed of cells toward anode. The influence of the ROCK blocker on the morphology of studied cells was also reported. ROCK was suggested to play a greater role in directing anodic motility under the electric field. In subsequent studies, Hou and collaborators [107] proposed a multichannel dual-electric-field (MDF) chip that permitted to simultaneously culture different types of cells in one experiment or simultaneously testing different chemical factors that may influence the electrotaxis.

Employment of dcEF for chemotactical CL1-5 cells modulation in a microfluidic cell culture chip was also performed by Kao et al. [77]. The results demonstrated that dcEF stimulation in the range of 180–540 mV mm^−1^ resulted in anode-directed migration of CL1-5 cells. Moreover, they postulated that EGF stimulation resulted in directional motion while dcEF stimulation gave rise to speed acceleration and direction of cells movement.

## Circulating tumor cells

Circulating tumor cells (CTCs) disseminate from the primary tumor. They intravasate into the circulatory system to travel to distant parts of the body organs in order to form the secondary tumor-metastatic disease [108]. The number of released tumor cells is unknown, however experimental data indicate that their number is extremely low, such as one CTC per million leukocytes [109] and one CTC per billion erythrocytes [110]. Moreover, they are highly heterogeneous and therefore may exhibit different phenotypes. Their properties may quickly evolve [111] contributing to difficulties in CTC isolation from whole blood. CTCs may also form CTCs aggregates that may provide valuable information about the nature of cancer metastasis. These are considered to be even 100-fold more metastatic in comparison to single CTCs [112].

The attempts of CTCs enrichment and isolation using microfluidic devices have been many times reviewed. Most of the microfluidic devices are constructed based on the biochemical and biophysical properties of CTCs. The biochemical properties of cells are used in affinity-based techniques. These techniques focus on the specific bonding between antigens expressed on the surface of cells and molecules fasten to various microstructures in the microfluidic system. Separation based on biophysical properties, named label-free isolation, mainly concerns the differences in size between CTCs and other components of blood, however, new size-independent approaches for electrical properties determination have been lately reported. In literature, we can even find the combination of aforementioned techniques [113]. Especially sensitive methods are necessary for CTC clusters isolation as they may detach into single cells or smaller aggregates. Hence, to preserve their integrity, isolation by size and asymmetry with application of low shear stress conditions were proposed [112, 114]. The advantages and disadvantages of both isolation techniques are summarized in Table 1 [42, 115, 116].

**Table 1:** Advantages and disadvantages of affinity-based and label-free isolation [17, 117, 118]

	Advantages	Disadvantages
Affinity-based isolation	Highly specific High purity of CTCs Application of the combination of antibodies allow to capture cells with epithelial and mesenchymal phenotype Capture of CTC clusters Separation/categorization based on abundance of Ep-CAM expression	Limitations in velocity and flow Blood volume Decrease of cell viability during the detachment process High cost of antibodies Low capture efficiency
Label-free isolation	High-throughput Allow CTCs capture regardless of subpopulation differences and EMT process Allow further cell molecular characterization Allow cell culturing and further recovery in suspension Allow preservation of cell clusters to study their metastatic ability Low cost	Capture depends on size pores Overlapping in CTCs and leucocytes sizes Pores clogging Low sample purity Low CTCs recovery

### Affinity-based techniques

Affinity-based techniques depend on highly specific reaction between cells’ surface specific antigens and ligands immobilized in the microfluidic device. The majority of the affinity-based methods utilize positive capture, i.e., trapping of CTCs via EpCAM—a transmembrane glycoprotein expressed on cancer cells’ surface [119]. The binding between EpCAM and the anti-EpCAM antibody coated on the microdevice channels is highly specific, however only enables to capture the CTCs that possess epithelial phenotype [120]. Such an approach is not appropriate for CTCs isolation that undergo an EMT process or express mesenchymal phenotype [121, 122]. More comprehensive applications exploit antibodies against the leucocyte antigen CD45 [123], called negative separation. This approach allows the isolation of all types of CTCs regardless of their phenotype. The application of antibodies against specific antigens toward particular cancers were also reported [115]. For example, anti-tyrosine-protein kinase receptor (anti-HER2) was employed for recognition of HER2 positive breast cancer [124]. An antibody for prostate-specific membrane antigen (PSMA) was used for prostate cancer circulating tumor cells entrapment [125]. Anti-podoplanin, an antibody against malignant pleural mesothelioma was also successfully used instead of anti-EpCAM [126].

Recently, aptamers, the single-stranded oligonucleotides (RNA and DNA), that possess the ability to specifically bind to proteins, ions or small molecules have drawn the attention to microfluidic systems constructors [127]. Aptamers are considered as better surrogates than antibodies as they are able to recognize more subtle features and low-immunogenic molecules of CTCs. The major advantages of aptamers are their high specificity and wide range of application regardless of specific knowledge about targeted molecule expression on the CTCs surface [128]. Recognition peptides are other linkers also acknowledged as adequate antibody substitutes for CTCs capture. This is due to their application simplicity and employment in ligand-receptor and protein–protein interactions [129].

The development of affinity-based techniques results in the improvement of the efficiency of CTCs separation from other components of blood and creation of the opportunity for cells segregation based on the abundance of surface markers expression [117, 130, 131]. This progress is possible among others through magnetic affinity-selection achieved by magnetic ranking cytometry (MagRC) [111, 132] and employment of magnetic items such as beads [17, 131, 133] and nanoparticles [17, 117, 130, 134, 135] coated with antibodies [131, 134], aptamers [17, 130] and peptides [121]. Employment of magnetic items permits the purification achievement by target cells gathering via a magnetic field [17], or by application of a magnetic sorter [131]. Magnetic ranking cytometry performs a nanoparticle mediated cells sorting in different zones according to their phenotypic ranking. The isolation undergoes through the binding of antibody-functionalized magnetic nanoparticles to surface expression markers of CTCs and subsequent cell separation according to a magnetic field gradient [111, 132]. However, recently, magnetic nanoparticles were also found to be employed for different CTCs subpopulation trapping [115].

The enhancement of capture efficacy can also be obtained by Fc-domain EpCAM antibody modification [120]. The capture yield can be increased by antibodies’ immobilization at various nanostructures such as nanopillars [15], nanofibers [136], nanorods [137] or nanowires [135]. They were reported to be composed or covered with gold nanoparticles [138], graphene oxide [122], or TiO_2_ [137] as they were accounted to augment the cells’ immobilization on various surfaces and enhance the cells’ capture. Carbon nanotubes [18] have also been used. The attempt of microbubbles decoration with EpCAM was also reported [139]. Equipment containing microfluidic chips with additional supporting units like multi-vortex mixing module [17] or X-shaped low flow regions [117] for increase of the effective binding were also notified.

Currently, microfluidic chips constructors endeavor to integrate all steps necessary for cells isolation, enrichment and detection into one chip. An example of such a device is the microfluidic system designed by Tsai et al. [17] that comprises RBC lysis, WBC depletion and CTC isolation. An interesting approach of CTC enrichment was proposed by Jiang et al. [118], whose microfluidic platform allowed the purification by sequential elimination of unwanted blood components by application of deterministic lateral displacement (DLD) and by automatic immunomagnetic purification [118].

### Label-free techniques

The advantage of label-free separation methods rely on the ability to isolate CTCs regardless of their surface markers, but with regard to cancer cells size [140, 141], fluid viscosity, cell density [113] and electrical properties [43, 142]. In the majority of label-free devices, the enrichment is achieved based on differences in physical properties between cancer cells and the other components of blood. The microfluidic methods developed for this purpose include a membrane-based separation [110], dielectrophoresis [143, 144], acustophoresis [113], and hydrodynamic-based separations [145]. Size-independent techniques like impedimetric separation are the latest achievements in the field of microfluidic systems progression [43, 142].

Membrane-based separation or filters employment depends on pores, gaps and microcavity array occurrence in devices’ construction. The membranes or filters possess pore/gap sizes from 5 µm up to 11 µm [115, 146], which were selected taking into account the cancer cells’ sizes and deformability. It was estimated that CTCs sizes are in the range of 10–20 µm, while red blood cells and leukocytes around 4–8.5 µm [116, 147] and 6–20 µm [116], respectively. However, CTCs from patients with non-small cell lung cancer were found to have a diameter range of 15–25 µm [148]. To separate cells with satisfactory efficiency the separation membrane should be made from appropriate materials. Membranes with suitable surface area, porosity and with satisfactory cell detachment were found to be created from polycarbonate [149, 150], parylene-C [151] and Mg-embedded parylene-C [152]. A membrane-based separation can be achieved among others by a double spiral microchannel application that hydrodynamically separates large CTCs from small blood cells [110] or centrifugal force application [150, 153]. Filters can be equipped with microcapillary arrays that may separate cells by deterministic lateral displacement. The significant impact on cells’ separation has also an assortment of appropriate flow rates. Low flow rates can lead to sample deterioration, however too high usually results in insufficient cell capturing as a result of passing through the microcavities [147, 154]. The flow rate cannot be clearly defined, its value depends on the shapes, sizes and microchannels geometry [155, 156]. Moreover, the flow rate may generate shear stresses and if these are high they may induce CTCs necrosis [157].

Hydrodynamic-based separations were recognized as highly throughput approaches. Currently, there is a significant number of microfluidic devices that were created based on hydrodynamic effects. They utilize the inertial fluid dynamic effects [16, 145], the Dean flow that occurs in spiral separators [110, 148, 158, 159], hydrophoresis [160], parallel multi-orifice flow fractionation (p-MOFF) [161], deterministic lateral displacement [162], and inertial separation [163].

The acustophoresis aims at cell specific lateral displacement based on cells’ acoustic mobility [164]. The separation is performed by a horizontal and vertical pre-alignment of the cells suspended in a sample by ultrasound. It allows cells’ location in a regular flow velocity regime within a parabolic flow profile [165]. This method enables cells’ differentiation based on their size, density, compressibility, or a combination thereof [166].

Dielectrophoresis allows to control cells’ movement in non-uniform electric fields based on their polarization and induction of a dipole moment [143, 167]. Wire electrodes are used to obtain the electric field, however recent reports suggest a more effective usage of arrays of wireless bipolar electrodes, since this overcomes cells’ capture and clogging in the channels and enables simultaneous entrapment across the parallel channels [168]. Recently, new microfluidic chips with optically induced dielectrophoresis were developed. The principle of the method is based on a uniform electric field generation for electrical polarization of microparticles. Subsequently, the light illumination of a photoconductive layer results in the generation of a non-uniform electric field. The interaction between the polarized electrical field and non-uniform electric field allows for microparticles’ manipulation for CTCs isolation from leukocytes based on the differences in their sizes [144, 169, 170]. Such methods are considered favorable for point-of-care applications [171].

**Fig. 4:**
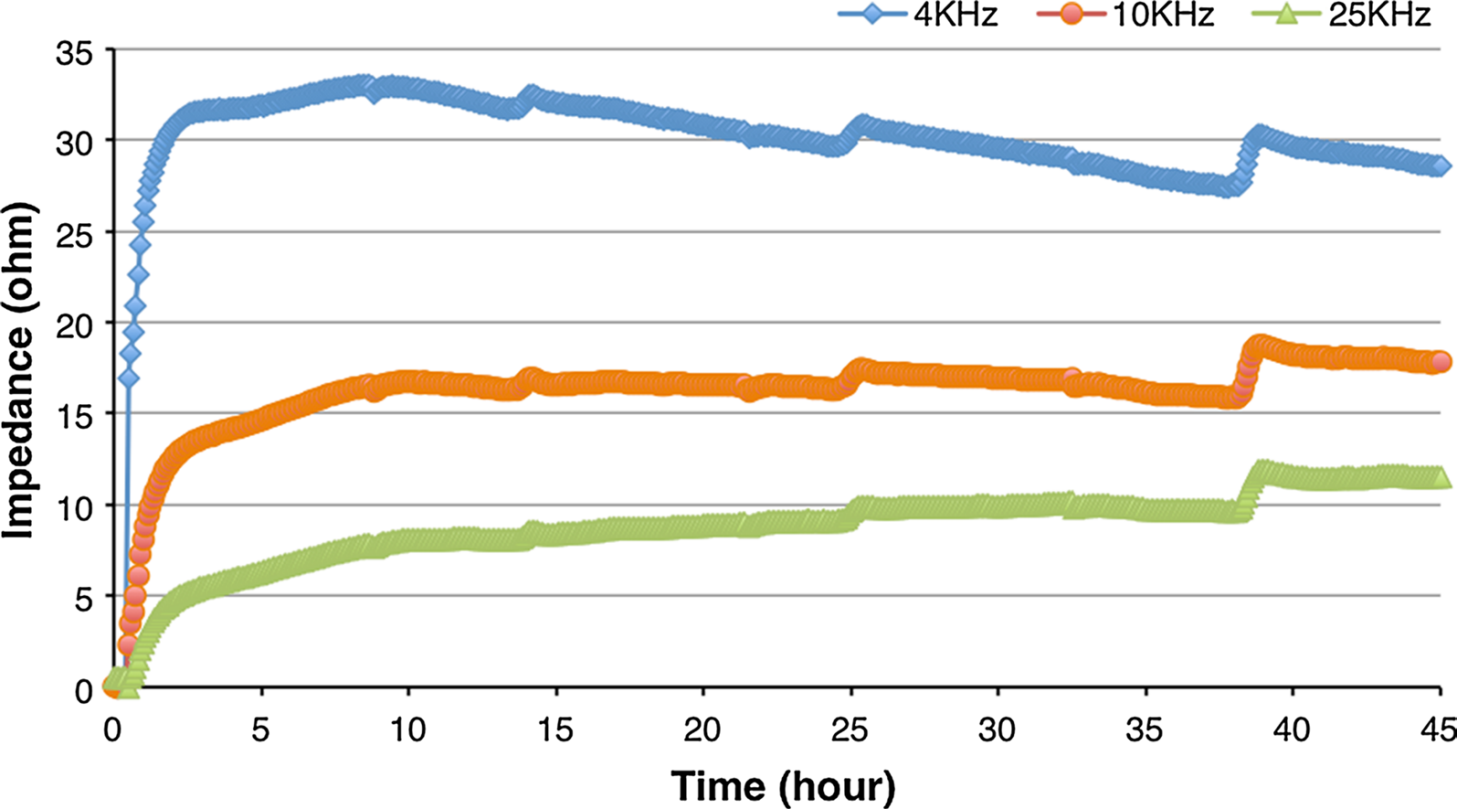
Impedance measurements of adenocarcinomic human alveolar basal epithelial cells (A549) in the microfluidic device originally designed in the “GEMNS” project (EuroNanoMed II program) by the Nanotoxicology group at the University of Bergen, Norway. Please see video information for A549 cells in the microfluidic device set-up (Additional file 1)

In recent times, size-independent techniques that allow CTCs and their subpopulation (epithelial- and mesenchymal CTCs) isolation using impedance [43, 142] were developed (Fig. 4). A device named nanoelectromechanical CTC Chip (NELMEC) was created for electrical signal measurement in order to distinguish entrapped cells (CTCs from large leucocytes) based on the differences in membrane capacitance [43]. Erythrocytes, platelets and small leucocytes freely pass through the channels and the cells’ entrapment takes place at the junction between microchannels and channels, where silicon nanograss (SiNG) electrodes are localized, which enable a direct electric signal measurement from captured cells. Another type of device is represented by the low-sample-loss microfluidic system that enables the characterization of CTCs’ electrical properties by measuring the specific membrane capacitance and cytoplasm conductivity [142]. The cellular electrical properties are measured using silver electrodes and a lock-in amplifier. An electrochemical impedimetric biosensor employed with multi-walled carbon nanotubes (MWCNTs) for CTCs detection was also constructed [18]. EpCAM antibodies were linked to MWCNTs which were assembled on indium tin oxide (ITO) glass, allowing an effective capture of cells on the electrode surface via binding with cell surface EpCAM. Impedance-based microfluidic flow cytometry (IBMFC) is also worth noting for CTCs identification [172]. As an example, the IBMFC can integrate a printed circuit board and a reusable pre-deposited copper electrode. Changes in the impedance signal between the electrodes carry the information about cell density, shape and morphological parameters.

Label-free CTCs separation usually needs further cellular refinement with immunostaining for CTC and leukocyte recognition [173], or gene identification by RNA-sequencing, qRT-PCR [174] or flow-cytometry [148] for quantification. This requires larger equipment and staff specialized in electronics, however these inconveniences are currently being addressed. A microchip that integrates optics for fluorescence quantification of CTCs has been described and the results obtained using the optofluidic device were consistent with those obtained from flow-cytometry, conventional imaging and serological tests [124].

### CTCs isolation from lung cancer metastasis patients

Several microfluidic systems have already been employed for lung metastasis screening of CTCs (Tables 2 and 3). They utilize anti-EpCAM antibody conjugation to magnetic upconversion of nanoparticles for CTCs capture [109], as well as anti-EpCAM substitution with A-1 peptide for different types of CTCs entrapment including those undergoing EMT process [121]. Parallel pre-EMT and post-EMT cells capture was also proven possible using nanoroughened glass [173]. This microfluidic platform for CTCs capture comprises two functional components: (i) a nanoroughened glass substrate with nanoscale topological structures to enhance adherent interactions between the glass substrate and cancer cells, and (ii) an overlaid PDMS chip with a low profile microfluidic capture chamber that promotes CTC-substrate contact frequency [173]. Immunostaining with cytokeratin-fluorescein isothiocyanate (FITC) for epithelial CTC [148], CD45- phycoerythrin (PE) for leukocyte [175], APC-conjugated anti-CD45 antibodies [148], and DAPI for nucleus [175], with Hoechst for DNA [148] can be be additionally employed. Usually the identification of CTCs is frequently associated with further refinement, e.g., by combination with genomic DNA extraction for mutational analysis of EGFR [176], with next generation sequencing [177], FISH assay application for identification of genomic alterations and mass spectrometry for mutational profiling [158] or loop-mediated isothermal amplification (LAMP) for detection of CK-19 mRNA from captured CTCs [110].

**Table 2:** Summary of analytical performance of microfluidic devices applied to real samples screening from lung cancer

Tumor type	Microfluidic device	Number of samples	Detection rate of CTCs	CTCs/ml (SD)	Additional tests	Refs.
NSCLC	CTC-Chip (affinity-based)	55 (human)	–	Mean: 155/ml (236)	EGFR mutation test	[176]
Lung cancer early stage patients	Immunoaffinity-based microfluidic device	19 (human)	68%	1–11/ml (3)	mRNA expression level determination	[177]
Cancer and non-cancer lung diseases	Microfluidic SiNW with MUNPs conjugated with anti-EpCAM	21 (human)	~ 90%	–	Immuno-fluorescence staining and imaging under the confocal fluorescence microscope	[109]
344SQ and 393P	Nanoroughened adhesion-based capture of CTCs	9 (mice)	Capture yields of > 80%	0–1148/ml (−)	Positive staining of anti-cytokeratin and DAPI; negative staining of anti-CD45; and appropriate morphometric characteristics including cell size, shape, and nuclear size	[173]
Lung cancer	Ultra-high throughput microfluidic Vortex technology	15 (human)	~ 80%	0.5–24.2/ml	Staining with DAPI, anti-CD45-PE, and FITC-conjugated CK cocktail against Pan-CK AE1/AE3, CK3-6H5, and CK CAM5.2 before imaging. Following CK staining, some samples were stained for granulocytes with CD66b-AlexaFluor647 (CD66b-AF647), or for EMT markers with anti EpCAM-FITC, anti-vimentin-AlexaFluor647 (VIM-AF647, Abcam), and anti N-Cadherin (NCAD-AF67, Abcam)	[175]
Advanced-stage metastatic non-small cell lung cancer	Ultra-high-throughput spiral microfluidic Biochip	5 (human)	100%	33–135/ml	Immunofluorescence staining and Fluorescent Automated Cytometry System (FACS) Analysis	[148]
Advanced stage metastatic lung cancers, patients with non-small cell lung cancer	Ultra High-Throughput Spiral Microfluidics	35 (human)	100%	10–1535/ml	Immunophenotyping (Pan-cytokeratin/CD45, CD44/CD24, EpCAM), FISH (EML4-ALK) or targeted somatic mutation analysis. Ultra-sensitive mass spectrometry based system to highlight the presence of an EGFR-activating mutation in both isolated	[158]
Metastatic lung cancer	Inertial-based microfluidic cell sorter	34 (human)	90%	–	Immunostaining and CK-19 mRNA detection	[110]
Lung cancer	FAST disc	35 (human)	68.6%	0–62/7.5 ml	Real-time polymerized chain reaction (PCR)	[150]
Lung cancer	Size-based microfluidic chip	77 (human)	–	1.85–68.45/ml	Immune-fluorescent staining combining an epithelial marker and a mesenchymal marker	[154]
Non-small cell lung cancer (NSCLC) patients	Label-free high-throughput microfluidic approach	16 stage IV NSCLC (human)	93.8%	–	Fluorescent staining (CK+/CD45-/DAPI+) and cytomorphological characteristics (large nuclear size > 9 μm and nuclear-to-cytoplasmic ratio > 0.8) to classify cells as CTCs	[178]
Lung cancer	Size-based microfluidic chip with contained array and filter channel array	200 (human)	Stage I (42.86%) Stage II (72.92%) Stage III (96.88%) Stage IV (96.49%)	Stage I (5.0 ± 5.121/ml) Stage II (8.731 ± 6.36/ml) Stage III (16.81 ± 9.556/ml) Stage IV (28.72 ± 17.39/ml)	Immunofluorescence staining, using epithelial marker (CK-FITC), DAPI and CD45-PE	[179]
NSCLC patients	Vortex HT chip	22 (human)	–	0.1 to 9.67 CTCs/ml	Immunostaining	[180]

*SiNW* silicon nanowire array, *MUNPs* multifunctional magnetic upconversion nanoparticles, *FAST* fluid assisted separation technology

**Table 3:** Summary of analytical performance of microfluidic devices applied to screen the sample spiked with lung cancer CTCs

Tumor type	Microfluidic device	Efficiency/capture rate	Additional tests	Refs.
A549	CTC-Chip (affinity-based)	87–100%	Immunofluorescence cell staining with CK7/8 or TTF-1 or Ki67 as well as the corresponding secondary antibodies. After FACS sorting, CTCs were stained with EGFR and pan-CK. CTCs also underwent RNA extraction, RT-PCR, TP53 sequencing and next-generation sequencing	[177]
A549	CTC-Chip (affinity-based)	60%	Cells were immunofluorescence (IF) stained for Cytokeratin 7/8 (green), white blood cells were stained for CD45 (red) and nuclei were counterstained with DAPI	[177]
A549	Microfluidic SiNW with MUNPs conjugated with anti-EpCAM	About 80%	Cells were stained with the method of immuno-fluorescence and then imaged under the confocal fluorescence microscope	[109]
A549	A-1 peptide modified microfluidic chip (affinity-based)	E-A549 (58.0 ± 19.7%) M-A549 similar to E-A549	The authors did not perform any additional tests	[121]
A549 and MDA-MB-231	Nanoroughened adhesion-based capture of CTCs	> 80%	CTCs were identified by: positive staining of anti-cytokeratin and DAPI; negative staining of anti-CD45; and appropriate morphometric characteristics including cell size, shape, and nuclear size	[173]
A549	Inertial‐based microfluidic cell sorter	74.4%	Loop-mediated isothermal amplification (LAMP) for detection of CK-19 mRNA from captured CTCs	[110]

*SiNW* silicon nanowire array, *MUNPs* multifunctional magnetic upconversion nanoparticles

## Organ on a chip

A continuous improvement and development of more and more sophisticated microfluidic systems is taking place in order to enhance the capacity to investigate highly complicated processes such a metastasis. Systems such as “organ on a chip” [181–183], “cancer on a chip” [184–186], and “metastasis on a chip” [187] etc. are among the latest achievements regarding biomimetics with regard to the functioning of living organs and providing appropriate conditions for studying the complex metastasis mechanisms. Employment of the dynamic microenvironment comprising multiple “organs”, vasculature network and CTCs in one chip allows the tracing and measurement of the metastatic potential of cancer cells [44].

Breathing lung-on-a-chip was engineered by Huh et al. [188]. This microdevice is composed of two superimposed flow-through microfluidic channels separated by a microporous membrane. The upper chamber (ventilation) supports the growth of human lung alveolar epithelial cells, while the lower chamber (perfusion) is lined with lung microvascular endothelial cells facing a constant flow of fluid to mimic the blood stream. To recapitulate the mechanical strain imposed by breathing movements, the cell culture chamber is flanked by two hollow microchannels trough which cyclic suction is applied causing expansion and relaxation of the membrane. This device could support the growth of microtumors derived from lung cancer cells to study cancer cell migration and therapeutic efficacy of aerosolized or infused nanotherapeutics. Microelectrodes can be also implemented to monitor in real-time transepithelial electrical resistance. This model offers the opportunity to study cancer cell migration under physiologically relevant conditions in vitro, which includes a multicellular context, as well as biochemical and mechanical cues (breathing motion and fluid perfusion). For instance, microtumors derived from lung cancer cells can be developed within the ventilation chamber to then study cancer cell migration and invasion of the endothelial barrier. The process of invasion can be monitored in real-time and in a label-free manner by using transendothelial electrical resistance. Microelectrode arrays could be also fabricated in one side of the microporous membrane to study in closer detail the process of invasion via electrical impedance spectroscopy. This model can not only be used to study the process of invasion, but also to test efficacy and possible side effects of novel chemotherapeutic agents administered as aerosols through the ventilation chamber or in solution via the perfusion chamber [188].

Xu et al. [189] have engineered a multi-organ microfluidic chip for replicating the complex lung and distant organs interactions. The device consisted of one upstream chamber devoted to lung imitation and 3 downstream chambers for brain, bone and liver recapitulation. The replication of the selected organs was achieved by bronchial epithelial, astrocytes, osteoblasts and hepatocytes seeding. The invasiveness of lung cancer cells was verified by measurement of RANKL for bone-specific metastasis, CXCR4 expression for brain-specific metastasis, and AFP for liver cell damage. The reliability of the results obtained from the application of a microengineered platform was confirmed by comparison with an in vivo model. The results obtained indicated that the proposed organ on chip model can be used for effective recapitulation of lung cancer cells’ metastasis to distant organs.

The utility of biomimetic microsystems for assessment of the metastatic potential of various cancers to lung metastasis was also demonstrated by Kong et al. [44]. The device allowed the modeling of the potential of breast and salivary gland cancer cells to metastasize lung, liver and bone marrow (muscle cells were used as a control). The results from clinical observations and the results from mentioned studies were found to be consistent, as the metastatic potential to lung, liver and bone marrow was significantly higher than to muscle cells. Simultaneously, they performed an in vivo study concerning lung metastasis on a nude mouse model. The obtained results from both studies revealed similar outcomes.

Besides “multi-organs” platforms that directly concern lung metastasis [189] or metastasis from lung [44], a type of platform called “metastasis on a chip” [187] should be noted. This was employed for the study of cells’ dissemination from colon carcinoma and their liver invasion and is not as advanced as the platforms aforedescribed, as it only possesses the possibility to study the interaction between two distinct tissues in the metastatic process.

**Fig. 5:**
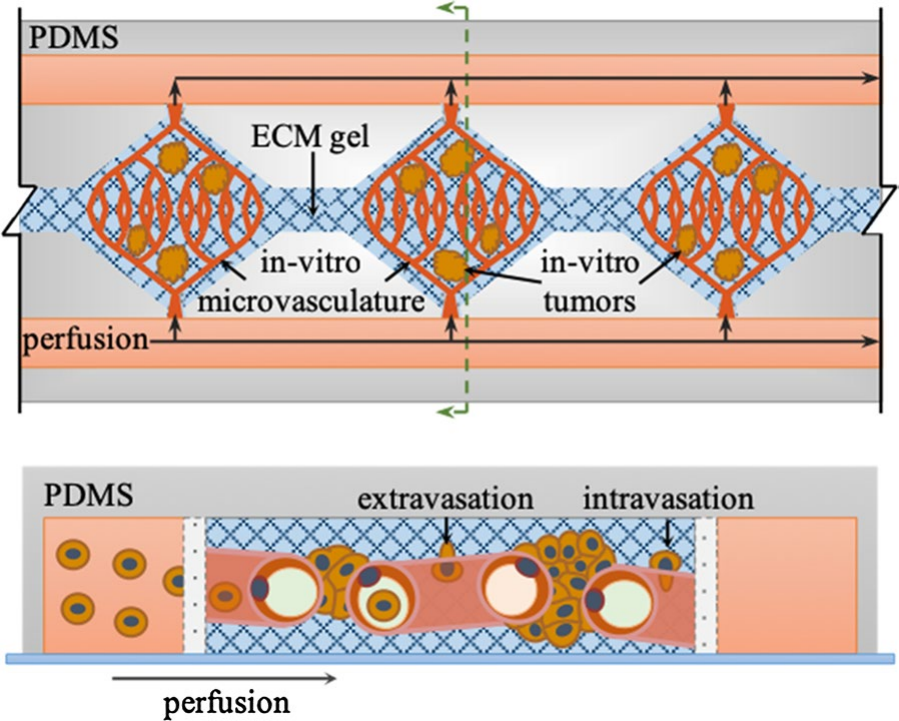
On-chip biomimetic model to study metastatic lung cancer. Tumor microvasculature-on-a-chip. Top-view (top) and cross section (bottom) of a multicompartment microfluidic chip for the development of perfusable microvascular networks and microtumors. Diamond-like chambers support the growth of microvascular networks emended in extracellular matrix (ECM) gels, while flanking side channels are used to perfused nutrients and drugs. Perfusable microvascular networks are formed by co-culturing microvascular endothelial cells with lung fibroblast and vascular smooth muscle cells in the ECM gel. Lung cancer cells can be co-injected before ECM gelification to grow microtumors. Alternatively, lung cancer cells can be perfused via flanking channels to study metastatic colonization (adapted from Sobrino et al. [190])

The tumor microvasculature model by Sobrino et al. [190] offers unique opportunities to study the processes of extravasation, intravasation and metastatic colonization for the formation of secondary tumors from lung CTCs (Fig. 5). This model relies on the development of fully perfusable microvasculature accompanying 3D microtumors embedded in ECM gels. Because these microvessels are perfusable, drug screening for cancer treatment can be seamlessly achieved in large-scale setups. The information obtained with this model can be used to develop new drugs that can more easily cross the endothelial barrier and the intricated ECM to effectively reach and act on the microtumors under hypoxic conditions. This model can be also used to study CTC extravasation for the design of drugs that block this process.

Besides metastasis investigation, attempts have been made for cancers’ recapitulation. One of the proposition is “tumor tissue-on-a-chip” developed by Astolfi et al. that used tumor cells directly obtained from biopsy or surgery [186]. The microfluidic chip had the potential of parallel screening of several drugs. It involved five microchannels with sedimentation trap for collecting the micro-dissected tumor/tissue samples (MDTs) from the specimen. The microchip permitted cells’ gathering in a constant number in each well with further submission to five different drug screening. The device proved to be suitable for the culturing of MDT cells from different types of patient tissues for several days and subsequent simultaneous cells’ observation after drug treatment. The usage of patient’s own cells for appropriate treatment selection could be an excellent solution for personalized treatment.

The whole human organs recapitulation on one miniaturized device is extremely important with regards to overcoming the shortcomings that arise from the imperfections and restrictions in application of in vivo, 2D and tissue 3D models [60]. The microfluidic-based organ-on-a-chip have numerous advantages, especially over static 2D models which provoke changes in cells morphology, their function and signaling preventing the cellular incorporation in a tumor structural conformation [191] and reproduction of the processes of tumorigenesis and metastasis. In 2D culture, cells are organized in a monolayer while in studies upon carcinogenesis and metastasis the formation of a multilayer tumor mass is fundamental for the recapitulation of intracellular crosstalk between cancer cells and ECM for subsequent initiations of cancer cells dissemination, migration and metastatic niche organization. Organ-on-a-chip in comparison to static models create the opportunity for different cell types interactions [44, 192] under controllable fluid and nutrients transfer imitating in vivo conditions. Moreover, the microfluidic chip can be a powerful tool to study of the effectiveness and toxicity of new therapeutics, which has many advantages over animal models, such as simplicity of use, time-saving and obtainment of fast results [192, 193]. Further improvements could result in diminishing the number of animals for testing and accelerate new drugs investigations [44].

## Theranostics and anti-cancer drug testing

Advances in the development of microfluidic systems enables the insightful analysis of molecular mechanisms of pathological processes and simultaneous novel therapeutic strategies implementation. The microfluidic devices have the ability to mimic the in vivo environment, which may be particularly significant during drugs testing, since over 90% of antitumor therapeutic agents with successful pre-clinical trials fail during human clinical testing [1, 194].

The rapid development in the field of microdevice engineering is evident, however most of the published research with usage of microfluidic devices is restricted to study of cells’ response to conventional chemotherapeutics, such as cisplatin, doxorubicin or paclitaxel. Conventional drugs generally have poor aqueous solubility, low bioavailability, insufficiency of selectivity toward cancerous cells and multidrug resistance [195]. In response to restrictions associated with conventional drug treatment, scientists undertook the challenge of breakthrough nanoparticles development that may act as therapeutics and diagnostic agents. The combination of high-throughput microfluidic devices with the possibility of testing multifunctional and precise theranostics will contribute to speeding up the time from development to implementation of new medicines and nanomedicines.

The development of nanotechnology provides the possibility to develop multifunctional nanostructures, aimed to be smart drug-delivery systems that combine the diagnostic and therapeutic features, called theranostics. The theranostic term was originally introduced by Funkhouser in 2002 [196]. Lately, much attention has been devoted to the development of nanoparticles for theranostics and to improvement of their properties, since they possess the potential for preferential drug delivery to cancer site and treatment monitoring [197]. One of the aims of theranostics is to augment the specificity of cancer treatment, forcing scientists to focus on targeted nanoparticles development. A wide range of potential theranostics has been reported including polymers [198], micelles [199], liposomes [200], quantum dots [201] and upconversion nanoparticles in their composition [202].

### Microchips in conventional chemotherapeutics testing

A wide array of microfluidic devices for high throughput drug screening have been reported [203]. Some of them have been used to test the effect of chemotherapeutics on various lung cancers. Zhao et al. constructed the microfluidic chip to investigate the impact of verapamil on P-glycoprotein (P-gp) expression [204] using immunofluorescence. Note that P-gp is responsible for diminishing the intracellular concentration of a broad range of cytotoxic agents, therefore resistance to the anticancer drug VP-16 after pretreatment of the human lung cancer cell line SPCA1 with verapamil or without was examined. The result suggested that verapamil could inhibit the P-gp expression contributing to enhancing the apoptosis induced by VP-16. The results were consistent with those obtained by flow cytometry.

One of the microfluidic device application in anticancer drug screening was demonstrated by Gao et al. [205]. They constructed a microfluidic system using photolithography which had HepG2 and A549 cells encapsulated in hydrogel microstructures, immobilized in microfluidic channels. The cytotoxicity assessed by measuring the level of intracellular glutathione (GSH) and of reactive oxygen species (ROS) in response to several concentrations of actinomycin D (Act D) and methotrexate (MTX). The presence of GSH and ROS was confirmed by fluorescence microscopy using labeling with 2,3-naphthalenedicarboxaldehyde (NDA) and dihydroethidium (DHE), respectively. The microfluidic system appeared to be a good replacement of traditional cells culturing in 96-well plate.

Xu et al. designed a microfluidic 3D co-culture drug sensitivity test platform [206] that aimed to construct an in vivo-*like* tumor environment to assess anti-cancer drug efficacy. The designed microfluidic system was equipped with four microfluidic units for simultaneous examination of four different cell cultures and five injection pump for flow rate monitoring. Each microfluidic unit consisted of three chambers for cell culturing, two inputs for drug introduction and fresh medium supply, and a concentration gradient generator for mixing of the content from both inlets. The drug sensitivity assay was performed on non-small the cell lung cancer cell line (SPCA-1) exposed to chemotherapy drugs: gefitinib, paclitaxel (PTX), and gemcitabine (GCB). The cytotoxicity of the studied chemotherapeutics was assessed with the methylthiazolyl-diphenyl-tetrazolium bromide (MTT) test. The microfluidic device occurred to be useful for the combined drug screening. Moreover, the results demonstrated the extreme significance of tumor stroma on drug efficacy, since the apoptosis rate in response of anticancer drug testing in SPCA-1 cells co-cultured with stromal cells diminished approximately two times in comparison with monoculture of SPCA-1 cells. The researchers also performed an individualized treatment on primary cells prepared directly from the fresh lung cancer tissues gained from patients. The results obtained on cells from fresh tissue differed from those obtained from cancer cell lines.

Another interesting approach for anticancer drug testing was presented by Dereli-Korkut et al. who constructed 3D microfluidic cell arrays for ex vivo drug screening with mimicked vascular flow [207]. The microengineered device had a three-layers construction for blood microvessels stimulation with an upper layer, permeable membrane with clustered pores in middle layer and 3D cell culturing in extracellular matrix in bottom layer. The experiment was carried out with usage of human ductal breast epithelial tumor cell line (T47D), human non-small cell lung cancer cell line (PC9), and adult human dermal blood microvascular endothelial cells (HMVEC) encapsulated in viscous liquid form of PuraMatrix hydrogel. The impact of apoptotic inducers such as tarceva, staurosporine, TNF-α, and colchicines on caspase-3 activities of PC9 cells was tested in conventional culture dishes and in the 3D microfluidic chip. Application of tarceva, staurosporine, and TNF-α with cycloheximide resulted in a fast increase of active caspase-3 in PC9 cells in a 12 h test, while colchicine stimulation gave greatly lower and slower response in time. Interestingly, at 17 h the highest caspase-3 activity was observed after staurosporine treatments. Beside this, the dynamic caspase-3 activity obtained from traditional 2D and microfluidic 3D testing were very different what may result from the absence of real circumstances prevailing in living organisms like shear stress applied by flow generation and waste products draining applied in microfluidic 3D chip.

A platform for mimicking organ-specific metastasis engineered by Kong et al. [44] was used for the anti-metastatic reagent AMD3100 testing in lung metastasis. In the study, AMD3100 had an inhibitory effect on CXCR12—chemokine supporting lung metastasis. Increasing concentrations of AMD3100 caused the reduction of primary lung cells and greatly inhibited lung metastasis of MDA-MB-231 cells in nude mice.

In another study, the impact of CAFs derived HGF on Met, PI3K and AKT phosphorylation, paclitaxel-induced apoptosis and glucose-regulated protein 78 (GRP78) expression in A549 cells was examined using of 3D microfluidic chip fabricated by Ying et al. [208]. The device consisted of two layers. The upper layer involved two inlets for medium and drug solution perfusion. The lower layer had an input for drug introduction, four parallel chambers for cells culturing and linear concentration gradient generator for diffusive mixing. Normal fibroblasts transformed to CAFs after exposition to A549 cells and HGF production by CAFs was evidenced. Moreover, CAFs occurred to up-regulated anti-phosphorylated Met, PI3K, AKT and anti-GRP78 activation which was subsequently abrogated by addition of human HGF neutralizing antibody. The treatment with paclitaxel revealed significantly lower A549 cell apoptosis when cells were maintained in a medium with CAFs than in non CAFs containing medium. Additionally, A549 cell apoptosis augmented during treatment with the inhibitor for PI3K or GRP78. All in all, the data obtained by researchers indicated that parallel inhibition of the PI3K and GRP78 improved lung cancer A549 cells apoptosis effect induced by paclitaxel.

## Microfluidics for nanosafety studies

### Microfluidics for toxicological and therapeutic testing of nanomaterials

**Fig. 6:**
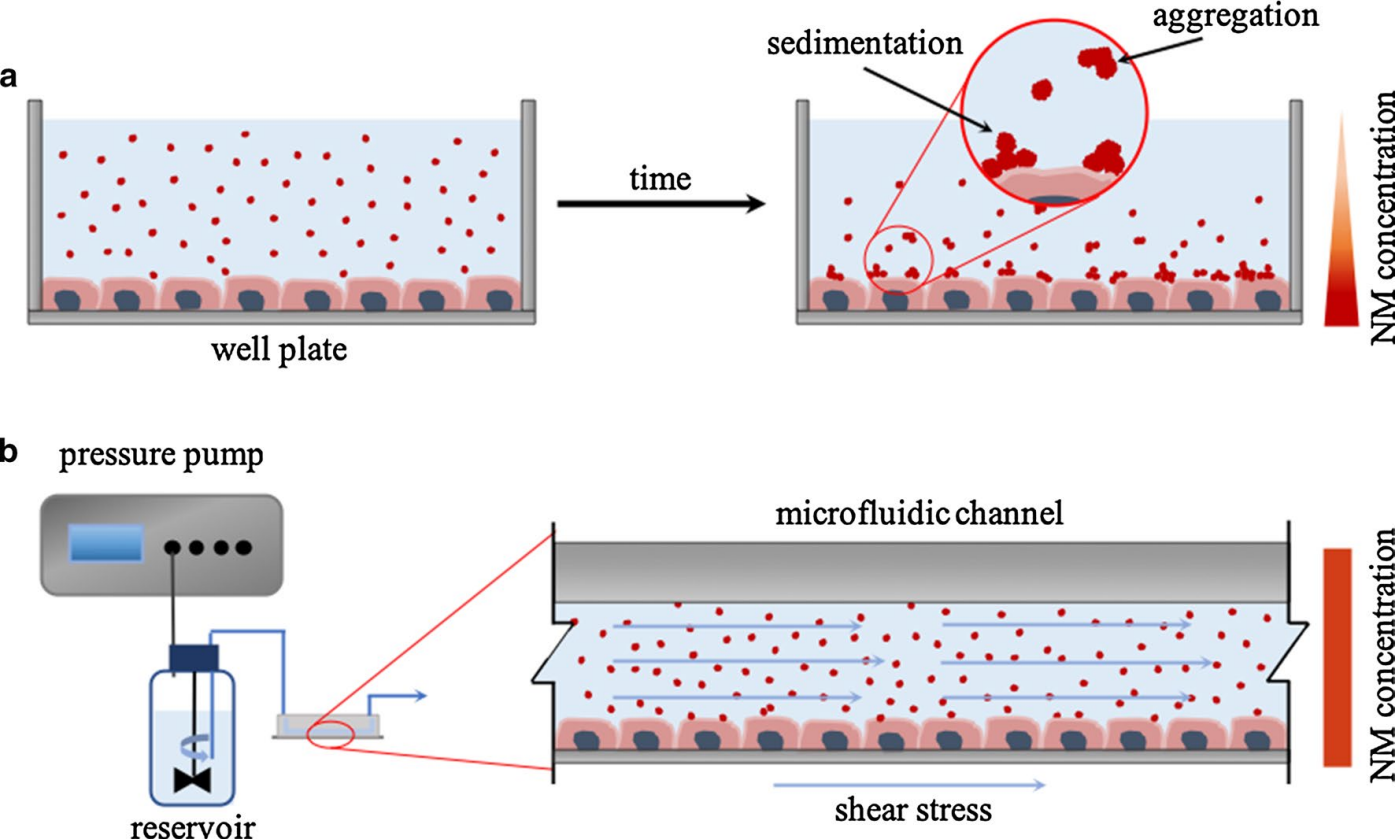
Static vs. dynamic conditions in cell-based assays for nanotoxicology. **a** Nanoparticles (NP)s tend to agglomerate and sediment in traditional cell-based assays performed under static conditions. This creates large particle agglomerates that are not readily taken up by cells. In addition, sedimentation generates concentration gradients. Therefore, delivered doses do not often match cellular doses (i.e., the amount of material in contact with and taken up by cells). **b** In contrast, cell-based assays performed in microfluidic devices, i.e., under dynamic conditions, allow perfusion of homogeneous NP dispersions from reservoirs equipped with mechanical stirrers. In addition, the fluid shear stress decreases NP agglomeration and sedimentation within the microfluidic channels. These two factors can be further reduced by designing microchannels structured with microgrooves and herringbone-microstructures to increase convective mixing

Traditional in vitro cell-based assays cannot accurately reflect the in vivo situation because they are commonly performed under non-physiologically relevant conditions. For example, traditional toxicological and therapeutic assays are performed under static conditions, which do not offer adequate control over cell exposure to nanomaterials (NM)s; i.e., agglomeration/aggregation, sedimentation and buoyancy, which impact the number and size of nanoparticles and their eventual agglomerates/aggregates that come in contact with cells [209]. In addition, static systems lack the dynamic microenvironment to which cells are exposed in in vivo systems. Within tissues, continuous perfusion of interstitial fluid provides fresh nutrients to cells and removes waste materials. This continuous fluid flow might impact the amount of NMs internalized by cells [210] (Fig. 6). Furthermore, conventional in vitro systems often fail to recapitulate the 3D structure of the microenvironment, cellular diversity and biomechanical cues of real tissue.

**Fig. 7:**
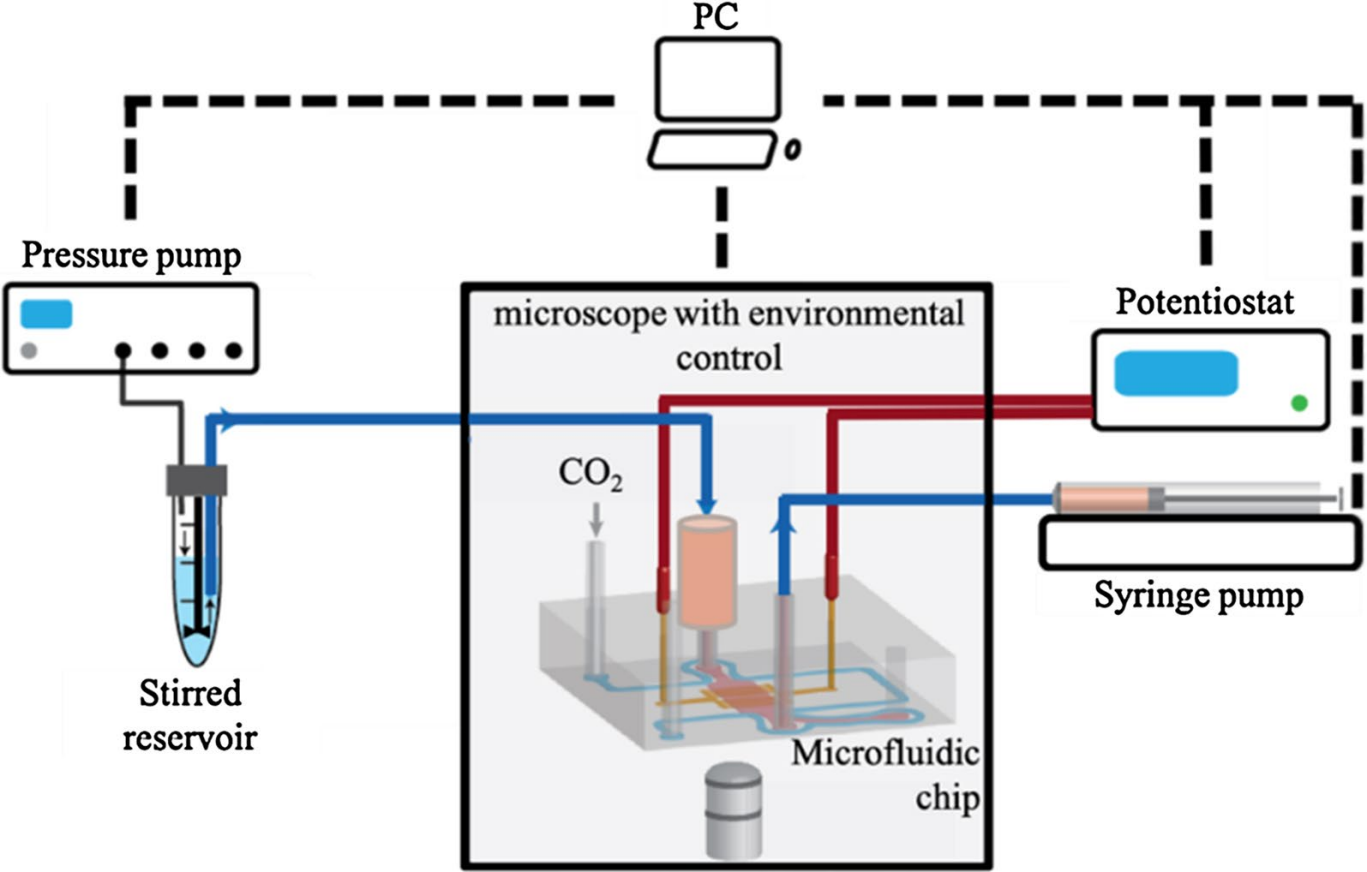
Schematic diagram depicting the components of the microfluidic platform developed in the GEMNS project (EuroNanoMed II program) by the Nanotoxicology group, University of Bergen, Norway. In this setup, custom-made on-chip reservoirs are directly attached to the chip inlets and the fluid is withdrawn continuously through the outlets using pulsatile-free syringe pumps. The on-chip reservoirs are automatically refilled with homogeneous nanoparticle dispersions every 30 min using a programmable pressure pump. To ensure dispersion homogeneity, the liquid reservoir of the pressure pump is kept under agitation using a magnetic stirrer with a stirring bar that does not interact with nanoparticles. The microfluidic device consists of four independent microfluidic chambers, each with a microelectrode array to evaluate the cytotoxicity via a cell-substrate impedance sensing. Impedance measurements were performed sequentially using an electrode switch and a potentiostat

Because of the limitations of conventional in vitro cell-based assays for NM testing, researchers have implemented microfluidic platforms that can more closely resemble real in vivo conditions (Fig. 7). Some of the advantages of microfluidic devices are: (1) well-controlled perfusion of homogeneously dispersed NPs, (2) continuous perfusion of fresh nutrients and removal of waste materials, (3) cell-volume to fluid ratio closer to physiological values, (4) fast and seamless switch between solutions to perform chronic and acute exposures, (5) precise delivery of reagents and NPs to sub-populations of cells, (6) precise cellular manipulation and positioning for patterning of multiple cell types in co-culture, (7) seamless generation of continuous concentration gradients of NPs, (8) generation of physiologically relevant mechanical stresses (e.g., shear forces) and (9) creation of dynamic and complex 3D environments.

Exposure to well-defined and homogeneous concentrations of nanomaterials is a paramount for reliable therapeutic and toxicological analyses [209]. In this regard, microfluidic platforms have been proven beneficial because the fluid drag force exerted on the nanoparticles circumvents particle sedimentation, buoyancy and agglomeration [211, 212]. Indeed, it has been demonstrated that quantum dot (QT)-induced cytotoxicity in fibroblasts is higher under static than flow exposure conditions. The authors of this study suggested that the settling down of QD over the cells might induce additional physicochemical stresses that could account for increased cytotoxicity. Similarly, HeLa cells responded differently when exposed to Ag NPs in static vs. perfusion conditions [210]. In this case, perfusion of Ag NPs lead to enhanced cytotoxicity by accelerating cell cycle arrest and subsequent apoptosis. Importantly, the cytotoxic effect augmented as the flow rate increased because of higher delivery of NP doses at faster flow rates with only a minor effect of the shear stress itself in cell viability.

Cytotoxicity of QDs has been also studied in neuron-like pheochromocytoma cells using a two-compartment microfluidic device [213]. In this set-up, axonal outgrowths were guided into a fluidically isolated compartment to study QD exposure locally in cell somas and axon terminals. Using this device, the authors found that cetyltrimethylammonium bromide/trioctylphosphine oxide-modified QDs were cytotoxic, causing cell shrinkage and axonal degeneration. Furthermore, selective exposure of cell somas and axon terminals to toxic QDs suggested that axonal degeneration could be a consequence of neuronal cell death and local axonal toxicity. This approach illustrates how microfluidic devices can be used to target subcellular compartments for nanotoxicological studies.

### Effect of shear stress in cellular uptake of NPs and toxicity

Endothelial cells (EC)s that line the luminal surface of blood and lymphatic vessels are constantly exposed to shear stress imposed by the flow of blood and lymph fluid, respectively. This mechanical stress acts on mechanosensitive receptors to regulate cell behavior [214]. In this regard, microfluidic devices have been widely used to study the response of a monolayer of ECs to shear stress and its influence on cellular uptake/interaction of NPs. For instance, ECs exposed to unmodified mesoporous silica NPs at different physiologically relevant shear forces (5–6 N m^−2^) exhibited increasing cytotoxic effects with increasing shear stress [215]. In contrast, highly organo-modified mesoporous silica NPs was not cytotoxic even under flow conditions. In this study, the NP concentration was adjusted for each shear stress level to match the NP dose of static condition and thus avoiding higher delivery of NP doses with higher flow rates. Therefore, the observed increase in cytotoxicity of unmodified silica NPs with higher shear forces was attributed to the flow shear stress acting on ECs to change their behavior. In fact, a later study demonstrated that ECs took up negatively charged CdTe QTs and silica nanoparticles under shear stress conditions, but not under static conditions within 20 min of NP exposure [216]. Interestingly, the authors of this study observed maximal cellular uptake of NPs at a shear stress value of 0.05 N m^−2^ and an increased number of cellular outgrows under shear stress conditions (e.g., filopodia and membrane ruffles) that may have facilitated the internalization of NPs. Fede et al. also demonstrated the relevance of shear stress for toxicological testing of Au NPs [217]. Here the authors observed that ECs exposed to citrate-stabilized Au NPs showed lower cytotoxic effects under flow conditions with respect to static exposure. Importantly, Fede et al. showed that the viability of ECs decreases with increasing total NP surface area per unit volume of solution, regardless of NP size.

Organic NPs such as lipidic, polymer- and protein-based NPs, have been proposed as drug delivery vehicles for treatment of several diseases. Although their potential as drug carriers is widely recognized (for a review see [218]), to date, only a few of these nanocarriers have been commercialized for intravenous drug delivery (e.g., Abraxane—protein-bound Paclitaxel, and Doxil—a liposomal formulation of doxorubicin). This highlights the need for relevant in vitro models for characterization and optimization of organic NPs as drug delivery vehicles.

An elegant study by Korin and coworkers demonstrated how to exploit high shear forces, similar to those experienced in obstructed blood vessels, to deliver drugs for rapid clot dissolution [219]. In this study, microscale agglomerates of NPs made out of poly(lactic-*co*-glycolic acid) (PLGA) were design to break up into nanoscale components when exposed to abnormally high shear stress. PLGA aggregates were stable in aqueous solution, but broke apart when exposed to high shear stress in a microfluidic device that served as model to mimic regions of living blood vessels with 90% of lumen obstruction. Moreover, released NPs accumulated in ECs lining the surface of the artificial microfluidic vessel distal to the constricted region with minimal uptake in cells located before the constriction. Upon validation in the microfluidic device, the authors investigated the therapeutic potential of NPs coated with the thrombolytic drug tissue plasminogen activator (tPA) in a mouse arterial thrombus model. In agreement with in vitro observations, tPA-coated NPs preferentially accumulated in regions of clot formation (i.e., high shear stress) and induced rapid clot dissolution, restoring normal flow dynamics [219].

Lipidic NPs have been also studied under shear stress conditions to modulate drug delivery and cell interaction. For example, shear-stress sensitive lentil-shaped liposomes were shown to release drugs locally at regions of elevated shear stress in a biomimicked model of atherosclerosis [220]. To enhance liposome interaction with ECs, liposomes can be functionalized with antibodies that recognize intercellular adhesion molecules expressed in ECs. For instance, Paulis and coworkers decorated liposomal MRI contrast agents with antibodies targeting the intercellular adhesion molecule-1 and analyzed their interaction with brain endothelial cells at physiologically relevant shear stress conditions [221]. The authors found that the higher the shear stress, the lower the efficiency of liposome-EC binding.

In a different study, Hosta-Rigau and Stadler made used of a microfluidic set-up to assess the response of mechanosensitive myoblast cells to liposomes, coated silica NPs and lipoplexes under static and low shear stress conditions (0.0146 N m^−2^) [222]. The authors showed enhanced interaction between positively charged liposomes and poly-l-lysine-coated silica NPs in the presence of low shear stress in comparison to static conditions. To note, irrespective of flow shear stress, zwitterionic and negatively charged liposomes poorly interacted with myoblast cells. To further demonstrate the relevance of shear forces for drug delivery, the authors loaded the positively charged liposomes with the hydrophobic antitumor compound thiocoraline and exposed the cells to this formulation in the presence of fluid flow. The results showed a circa 50% decrease in cell viability when thiocoraline-loaded liposomes were administered in the presence of low shear stress as compared to static conditions. Finally, this study also demonstrated that cationic lipid-mediated gene delivery was more efficient when myoblast cells were exposed to shear stress than in static culture conditions. A similar study by Teo et al., demonstrated no significant difference in the uptake of PEGylated positively charged or non-PEGylated liposomes by myoblasts under static and flow conditions [223]. By contrast, liposome uptake by hepatocytes was significantly higher for non-PEGylated than PEGylated liposomes when exposed to shear stress. These results demonstrate the relevance of shear stress for studying the interaction of NPs not only on ECs, but also with other cell types that may come into contact with NPs because of biodistribution.

Taken together, all these studies emphasize the relevance of microfluidic devices to biomimic the dynamic environment to which cells are exposed in vivo for reliable assessment of cytotoxic effects of NPs. They also demonstrate that blood flow-induced shear forces have to be considered for the design of nanocarriers for drug delivery and that microfluidic devices are ideal for biomimicking this scenario.

### Studying NP-cell interactions in microengineered biomimetic models

Organs-on-chips are microengineered cell culture models that replicate cellular composition, microenvironment architecture, mechanical cues and physiological function of key units of living human organs. Consequently, organs-on-chips are now being implemented as means to obtain reliable predictions of drug efficacy and NP cytotoxicity. Pioneering work by Huh et al., demonstrated the use of a lung-on-a-chip model for nanotoxicological testing under physiologically relevant conditions [188]. They microengineered the alveolar-capillary interface in a microfluidic device by co-culturing human alveolar epithelial and human pulmonary microvascular endothelial cells in opposite sides of a thin porous elastomeric membrane. The membrane served as the interface between the upper alveolar and lower microvascular chambers of the microfluidic device. Furthermore, mechanical actuation of the elastomeric membrane was implemented to mimic physiological breathing motions. This biomimicking approach revealed that cyclic mechanical strain enhances toxic and inflammatory responses of the lung to silica NPs. In addition, mechanical strain enhanced the cellular uptake of NPs by epithelial and endothelial cells, which facilitated the transport of NPs into the lower microvascular chamber. Interestingly, silica NPs and Cd/Se QDs induced the production of reactive oxygen species in both cellular layers under mechanical stress. By contrast, superparamagnetic Fe NPs, Ag NPs, polystyrene NPs, singled-wall carbon nanotubes and PEG-coated QDs did not induce an oxidative response.

In a later study, Kim and coworkers developed a blood vessel-on-chip model to study NP translocation across a dysfunctional endothelial barrier in experimental atherosclerosis [224]. Their device consisted of an upper and lower microfluidic chamber interfaced by a thin semiporous membrane. ECs were grown in the upper chamber and a leaky endothelial barrier characteristic of atherosclerosis was induced by perfusion of TNFα and low shear stress (< 0.4 N m^−2^). Under these conditions, PEGylated liposomes readily translocated across the dysfunctional endothelial barrier. Importantly, the results obtained with the microfluidic devices were corroborated by data from animal models.

NP transport from capillaries into surrounding tissues has been also addressed using biomimetic microfluidic devices. For instance, Wu et al. fabricated a device comprising a main chamber, representing a blood vessel, and two sets of side cell culture chambers. The main and side chambers were interconnected by stop-flow junctions that only permit mass transport by means of diffusion. In this device, human hepatocellular liver carcinoma cells were grown in the side cell culture chambers embedded in an agarose matrix and QDs were perfused in the main chamber. The results of this study revealed a dose-dependent increase in the cytotoxic effects of QDs. In addition, it was shown that cell autophagy played a key role in QD cytotoxicity.

Incorporating tumor spheroids into microfluidic devices will help to better understand NP penetration into solid tumor tissue and thus improve NP design for enhanced drug delivery. In this regard, Albanese and coworkers developed a tumor-on-a-chip system consisting of a multicellular spheroid immobilized within a microfluidic channel that allowed continuous perfusion of Au NPs [225]. The spheroid was composed of melanoma cells embedded in an extracellular matrix forming a tortuous network of interstitial spaces. The authors showed that penetration of NPs into the tumor tissue is diffusion-limited and dependent on NP size; while NPs < 110 nm can diffuse through the ECM and interact with melanoma cells, NPs > 110 nm mainly localize to the periphery of the spheroid. In addition, NP retention within the tumor interstitium can be improved by receptor targeting. Notably, a murine tumor model corroborated the findings from the tumor-on-a-chip device.

## Future perspectives

Microfluidic systems have a potential, by enabling earlier cancer molecular diagnosis, for a better targeted cancer therapy and improved follow-up care, to make the care process more effective in terms of clinical outcome for cancer patients’ needs. The application of microfluidics to novel diagnostics and anticancer drug design has demonstrated a significant impact on many areas of modern nanomedicine. It is becoming increasingly more evident from the number of ongoing research programs that microfluidic systems play a pivotal role in personal nanomedicine. Perhaps one of the essential areas of the research in personalized nanomedicine to move forward is the development of the point-of-care (POC) diagnostics based on novel microfluidic platforms to help assign particular early molecular diagnostics and target treatment schedules to individuals with diseases such as lung cancer. Therefore, microfluidic devices will be further developed from the bench-to-bedside and updated to produce patient-friendly analytical assays. Impedance-based measurements could be applied in microfluidic systems to enable a quick screening of single lung cancer cells in order to identify their metastatic potential. To speed up the analysis and make it patient friendly, the so-called paper-based microfluidic devices and lab-on chips based on paper imprinting technology will be developed [226]. Their real advantage will be the simplicity of design and ease of interpretation of test results regarding POC diagnostics [227]. Combining both microfluidic and bioprinting technology will constitute growing areas in upcoming future. Such bioprinting technologies will be improved by bioengineering of 3D constructs that mimic tumor heterogeneity, vasculature and spheroid structures. Furthermore, bioprinting processes will be used to fabricate cancer tissue constructs within microfluidic platforms, forming lung tumor-on-a-chip devices which are ideal for high-throughput testing in a biomimetic microenvironment.

Microfluidic devices for assessing the impact of the surrounding microenvironment and circulating tumor cells detection have been widely described in the literature. The progress of the latter is extremely dynamic due to their diagnostic potential and the possibility to use as POC. The progress in microengineering manifests also in more comprehensive devices that recapitulate organs and cancers’ environments in vivo, processes like metastasis and carcinogenesis allow the testing of conventional anticancer drugs, theranostics and nanoparticles as well as studying the nanosafety. Although, the development in microfluidic devices is going fast, the ideal microfluidic system that would perfectly imitate in vivo metastasis conditions has not yet been created, hence there are still limitations that need to be addressed. Modern microfluidic chips are equipped with automated continuous flow running of biofluid, stable gradient maintenance and continuous digital cell behavior registration [78]. Microfluidic devices with a greater potential to reproduce in vivo conditions possess biocompatible membranes with pores [76] and/or other microunits that may constitute the scaffold for endothelium [76] aimed at building up real structures occurring in organism.

**Fig. 8:**
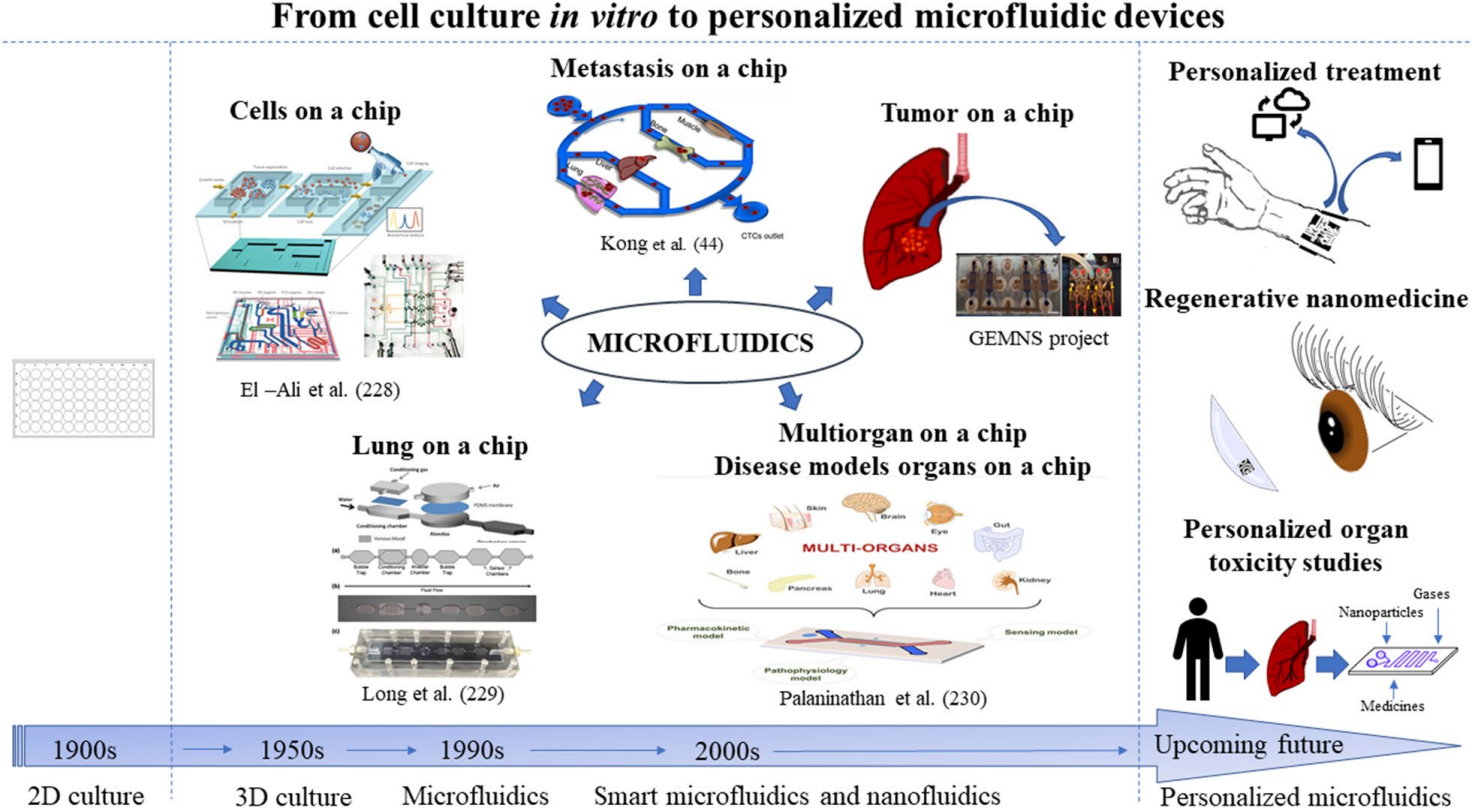
Recent progress of microfluidic technologies in nanomedicine. The figures are reproduced from El-Ali et al. [228] with permission of Nature, Kong et al. [44] with the permission of Oncotarget, Long et al. [229] with the permission of Annals of Biomedical Engineering, Palaninathan et al. [230] with permission of MRS Communication

In order to improve currently existing microfluidic devices, aspects like (1) imitation of in vivo tumor conditions, (2) automatization and digitization of essential components of medium supply, and (3) microdevice with integrated functions of analytical and omics techniques still need to be considered. In order to create in vivo conditions an adequate material for microfluidic construction need to be selected. The stiffness of the material as well as its biocompatibility will affect cells culturing. The spatial construction of a device should enable cells culturing in multilayers for accurate mimicking of a solid mass tumor. A spherical structure of a tumor reproduced by cells in artificial conditions provides a recapitulation of cells’ morphology, cell–cell and cell-ECM interactions which have a great impact on differentiation, proliferation, expression the genes and proteins, response to stimuli, drug metabolism and other cellular functions [191]. The selection of appropriate biofilm like gel or culturing in a scaffold is essential for 3D cells conformation establishment [191]. In order to study metastasis, the tumor cells need to interact with different tissues like (a) the epithelium layer (necessary for transendothelial cell migration), (b) potential places of metastatic niche development. The above aim would among others be obtained with the aid of bioprinting technologies 3D constructs that mimic tumor heterogeneity, vasculature and spheroid structures. Cells, as well as developing tumors, need a constant and controllable supply of oxygen, nutrients, metabolites and signal molecules in a biofluid under defined shear stress [191], as well as drain of toxic metabolic products. Those processes need to be automatized. The investigation of the biochemistry of the tumor needs working with chemical gradients and electrical stimuli that also should be automatized in order to be stable and reproducible. Finally, microdevices should be equipped with constant digital registration of cells’ behavior. Creation of program for cell type identification would enable resignation from cells staining and fixing allowing their continuous tracking. In the study upon metastasis it is also important to apply “from the bench-to-bedside” rule. Efforts have been put into in vivo conditions reconstruction into one chip, however there is also a possibility to use similar chips in human organism. For example, a chip that serves for CTCs detection could be installed into the body of a person with cancer suspicion or recurrence. After the detection of CTC the microfluidic device would automatically send the information to other electronic devices (Fig. 8). Other possibility is patient friendly, paper-based microfluidic device that could serve for lung cancer determination. There is also a need to deeply develop microfluidic devices for personalized toxicity studies. The proposition is even more actual in a view of growing air pollution and spreading smog responsible for lung disease including cancer. In this case it would be even a life-saving device.

The miniaturization of analytical and omics techniques has been previously predicted [1], however there is still a need of incorporation all these techniques into single microdevice. Progress in this field is noticeable since microdevices for DNA nanotechnology [231] and proteomic and metabolomic profiling [232] have been recently developed, however for maintaining reproducibly and repeatability of the results and eliminating cells contamination and errors related to material processing all techniques should be combined in a way of merging cell handling with analytical, biochemical and molecular determination.

Improving the ability to predict the efficacy and toxicity of new anticancer drugs including theranostic nanomaterials earlier in the drug discovery process will speed up the introduction of novel drug candidates into clinical trials. Note that 3D in vitro systems placed on microfluidic devices will significantly support the new drug screening process as the 3D tissue cancer models can closely mimic the native tissues and, in some cases, the physiological response to novel drug molecules [233]. Therefore, microfluidic systems will be an essential approach to address many unmet lung cancer diagnostic and therapeutic needs in the coming future.

## Additional file

**Additional file 1.** Handling of A549 cells in the microfluidic device developed by the Nanotoxicology group at the University of Bergen, Norway.

## Data Availability

Not applicable.

## Abbreviations

2D: two-dimensional
3D: three-dimensional
Act D: actinomycin D
Angpt2: angiopoietin 2
Angptl4: angiopoietinlike-4
Anti-HER2: anti-tyrosine-protein kinase receptor
AFP: α-fetoprotein
BME: Basement Membrane Extract
BMECs: brain microvascular endothelial cells
CAFs: cancer-associated fibroblasts
CM: collagen:Matrigel
CTCs: circulating tumor cells
CXCR4: chemokine receptor type 4
dLCSC: differentiated lung cancer stem cell
DHE: dihydroethidium
DLD: deterministic lateral displacement
ECM: extracellular matrix
EGF: epidermal growth factor
EMT: epithelial–mesenchymal transition
FBS: fetal bovine serum
ERK: extracellular signal-regulated kinase
FITC: cytokeratin-fluorescein isothiocyanate
GCB: gemcitabine
GSH: glutathione
GRP78: glucose-regulated protein 78
HMVEC: human dermal blood microvascular endothelial cells
IBMFC: impedance-based microfluidic flow cytometer
ITO: indium tin oxide
LAMP: loop-mediated isothermal amplification
LCSC: lung cancer stem cell
NSCLC: non-small cell lung cancer
N-WASP: neural Wiskott–Aldrich syndrome protein
M1: normal macrophages
M2: tumor-promoting macrophages
MagRC: magnetic ranking cytometry
MMPs: matrix metalloproteases
MTT: methylthiazolyl-diphenyl-tetrazolium bromide test
MTX: methotrexate
MWCNTs: multi-walled carbon nanotubes
NELMEC: nanoelectromechanical CTC Chip
NDA: 2,3-naphthalenedicarboxaldehyde
NMs: nanomaterials
NPs: nanoparticles
PKC: protein kinase C
PDGF: platelet-derived growth factor
PDMS: polydimethylsiloxane
p-MOFF: parallel multi-orifice flow fractionation
PCR: polymerase chain reaction
P-gp: P-glycoprotein
PMNs: pre-metastatic niches
PSMA: prostate specific membrane antigen
PTEN: phosphatidylinositol-3,4,5-trisphosphate 3-phosphatase
PTX: paclitaxel
QT: quantum dot
RANKL: Receptor Activator for Nuclear Factor κ B Ligand
ROS: reactive oxygen species
RTK: receptor tyrosine kinase
SCLC: small cell lung cancer
SiNG: silicon nanograss
TAMs: tumor-associated macrophages
TGFβ: transforming growth factor β
t-PA: tissue plasminogen activator
YBX1: Y-box-binding protein-1

## References

[1] Whitesides GM (2006). The origins and the future of microfluidics. Nature.

[2] Eijkel JCT, Berg AVD (2005). Nanofluidics: what is it and what can we expect from it?. Microfluidics Nanofluidics.

[3] Chin CD, Linder V, Sia SK (2012). Commercialization of microfluidic point-of-care diagnostic devices. Lab Chip.

[4] Sackmann EK, Fulton AL, Beebe DJ (2014). The present and future role of microfluidics in biomedical research. Nature.

[5] Anguiano M, Castilla C, Maska M, Ederra C, Pelaez R, Morales X (2017). Characterization of three-dimensional cancer cell migration in mixed collagen-Matrigel scaffolds using microfluidics and image analysis. PLoS ONE.

[6] Gupta N, Liu JR, Patel B, Solomon DE, Vaidya B, Gupta V (2016). Microfluidics-based 3D cell culture models: utility in novel drug discovery and delivery research. Bioeng Transl Med.

[7] Sosa-Hernández JE, Villalba-Rodríguez AM, Romero-Castillo KD, Aguilar-Aguila-Isaías MA, García-Reyes IE, Hernández-Antonio A (2018). Organs-on-a-chip module: a review from the development and applications perspective. Micromachines..

[8] Li Y, Gao A, Yu L (2016). Monitoring of TGF-beta 1-induced human lung adenocarcinoma A549 cells epithelial-mesenchymal transformation process by measuring cell adhesion force with a microfluidic device. Appl Biochem Biotechnol.

[9] Wang S, Li E, Gao Y, Wang Y, Guo Z, He J (2013). Study on invadopodia formation for lung carcinoma invasion with a microfluidic 3D culture device. PLoS ONE.

[10] Breiman A, Lopez Robles MD, de Carne Trecesson S, Echasserieau K, Bernardeau K, Drickamer K (2016). Carcinoma-associated fucosylated antigens are markers of the epithelial state and can contribute to cell adhesion through CLEC17A (Prolectin). Oncotarget.

[11] Zhao H, Wang J, Kong X (2016). cd47 promotes tumor invasion and metastasis in non-small cell lung cancer. Sci Rep..

[12] Li XJ, Valadez AV, Zuo P, Nie Z (2012). Microfluidic 3D cell culture: potential application for tissue-based bioassays. Bioanalysis..

[13] Kim SH, Hwang SM, Lee JM, Kang JH, Chung IY, Chung BG (2013). Epithelial-to-mesenchymal transition of human lung alveolar epithelial cells in a microfluidic gradient device. Electrophoresis.

[14] Bai J, Adriani G, Dang T-M, Tu T-Y, Penny H-XL, Wong S-C (2015). Contact-dependent carcinoma aggregate dispersion by M2a macrophages via ICAM-1 and β2 integrin interactions. Oncotarget.

[15] Wang S, Wan Y, Liu Y (2014). Effects of nanopillar array diameter and spacing on cancer cell capture and cell behaviors. Nanoscale..

[16] Ramachandraiah H, Svahn HA, Russom A (2017). Inertial microfluidics combined with selective cell lysis for high throughput separation of nucleated cells from whole blood. RSC Adv.

[17] Lee TY, Hyun KA, Kim SI, Jung HI (2017). An integrated microfluidic chip for one-step isolation of circulating tumor cells. Sens Actuators B Chem.

[18] Liu Y, Zhu F, Dan W, Fu Y, Liu S (2014). Construction of carbon nanotube based nanoarchitectures for selective impedimetric detection of cancer cells in whole blood. Analyst.

[19] Chen MB, Whisler JA, Fröse J, Yu C, Shin Y, Kamm RD (2017). On-chip human microvasculature assay for visualization and quantification of tumor cell extravasation dynamics. Nat Protoc.

[20] Grand View Research. Microfluidics market size, share & trends analysis report by application (pharmaceutical, in vitro diagnostics, by material, by region, and segment forecasts, 2018–2024); 2018. https://www.grandviewresearch.com/industry-analysis/microfluidics-market. Accessed 1 Mar 2019.

[21] Markets and Markets. Microfluidics market worth 27.91 Billion USD by 2023; 2018. https://www.marketsandmarkets.com/PressReleases/microfluidics.asp. Accessed 1 Mar 2019.

[22] Markets and Markets. Microfluidics market by application, component and material—global forecast to 2023; 2018. https://www.researchandmarkets.com/research/km2gn7/microfluidics?w=4. Accessed 1 Mar 2019.

[23] Brzozka Z, Jastrzebska E (2017). Cardiac cell culture technologies: microfluidic and on-chip systems.

[24] Hu G, Li D (2007). Multiscale phenomena in microfluidics and nanofluidics. Chem Eng Sci.

[25] Merck. Microfluidic plate. http://www.merckmillipore.com. Accessed 1 Mar 2019.

[26] Ibidi. *µ*-Slide III 3in1. https://ibidi.com. Accessed 1 Mar 2019.

[27] Darwin_microfluidic. https://darwin-microfluidics.com/. Accessed 1 Mar 2019.

[28] Micronit. https://www.micronit.com/. Accessed 1 Mar 2019.

[29] uFluidix. https://ufluidix.com/. Accessed 1 Mar 2019.

[30] Elveflow. HOME Microfluidic flow control. https://www.elveflow.com/. Accessed 1 Mar 2019.

[31] Micralyne. MEMS foundry—MEMS manufacturer & MEMS development. https://www.micralyne.com/. Accessed 1 Mar 2019.

[32] Dolomite. Microfluidic systems and components 2018. https://www.dolomite-microfluidics.com/.Accessed 1 Mar 2019.

[33] PRNewswire. Microfluidics market by material (ceramics, polymers), by components (microfluidic chips, pumps, needles), by application (in-vitro diagnostics, pharmaceutical research, drug delivery)—world forecasts to 2022; 2018. https://www.prnewswire.com/news-releases/microfluidics-market---by-material-ceramics-polymers-by-components-microfluidic-chips-pumps-needles-by-application-in-vitro-diagnostics-pharmaceutical-research-drug-delivery--world-forecasts-to-2022-300713744.html. Accessed 1 Mar 2019.

[34] Mordor Intelligence. Microfluidics market—growth, trends, and forecast (2019–2024); 2019. https://www.mordorintelligence.com/industry-reports/microfluidics-market. Accessed 1 Mar 2019.

[35] Wu WI, Rezai P, Hsu HH, Selvaganapathy PR (2013). 1—Materials and methods for the microfabrication of microfluidic biomedical devices. Microfluidic devices for biomedical applications.

[36] Cho SK, Zhao Y, Kim CJ (2007). Concentration and binary separation of micro particles for droplet-based digital microfluidics. Lab Chip.

[37] Voldman J, Gray ML, Schmidt MA (1999). Microfabrication in biology and medicine. Annu Rev Biomed Eng.

[38] Tata U, Rao SM, Sharma A, Pabba K, Pokhrel K, Adhikari B (2012). Study of lung-metastasized prostate cancer cell line chemotaxis to epidermal growth factor with a BIOMEMS device. Adv Nat Sci Nanosci Nanotechnol.

[39] Huang CW, Cheng JY, Yen MH, Young TH (2009). Electrotaxis of lung cancer cells in a multiple-electric-field chip. Biosens Bioelectron.

[40] Zhu S, Li H, Yang M, Pang SW (2018). Label-free detection of live cancer cells and DNA hybridization using 3D multilayered plasmonic biosensor. Nanotechnology..

[41] Huang XJ (2008). Nanotechnology research: new nanostructures, nanotubes and nanofibers.

[42] Murlidhar V, Rivera-Baez L, Nagrath S (2016). Affinity versus label-free isolation of circulating tumor cells: who wins?. Small.

[43] Hosseini SA, Abdolahad M, Zanganeh S, Dahmardeh M, Gharooni M, Abiri H (2016). Nanoelectromechanical chip (NELMEC) combination of nanoelectronics and microfluidics to diagnose epithelial and mesenchymal circulating tumor cells from leukocytes. Small.

[44] Kong J, Luo Y, Jin D, An F, Zhang W, Liu L (2016). A novel microfluidic model can mimic organ-specific metastasis of circulating tumor cells. Oncotarget..

[45] Huh D, Torisawa Y-S, Hamilton GA, Kim HJ, Ingber DE (2012). Microengineered physiological biomimicry: organs-on-chips. Lab on a Chip..

[46] Luni C, Serena E, Elvassore N (2014). Human-on-chip for therapy development and fundamental science. Curr Opin Biotechnol.

[47] Zhao H, Wang J, Kong X, Li E, Liu Y, Du X (2016). CD47 promotes tumor invasion and metastasis in non-small cell lung cancer. Scientific Rep.

[48] Molina JR, Yang P, Cassivi SD, Schild SE, Adjei AA (2008). Non-small cell lung cancer: epidemiology, risk factors, treatment, and survivorship. Mayo Clin Proc.

[49] Yuan P, Cao JL, Rustam A, Zhang C, Yuan XS, Bao FC (2016). Time-to-progression of NSCLC from early to advanced stages: an analysis of data from seer registry and a single institute. Scientific Rep.

[50] Pinchot SN, Holen K, Sippel RS, Chen H (2008). Carcinoid tumors. Oncologist.

[51] Riihimaki M, Hemminki A, Fallah M, Thomsen H, Sundquist K, Sundquist J (2014). Metastatic sites and survival in lung cancer. Lung cancer (Amsterdam, Netherlands)..

[52] Ngan E, Stoletov K, Smith HW, Common J, Muller WJ, Lewis JD (2017). LPP is a Src substrate required for invadopodia formation and efficient breast cancer lung metastasis. Nat Commun.

[53] Villeneuve PJ, Sundaresan RS (2009). Surgical management of colorectal lung metastasis. Clin Colon Rectal Surg.

[54] Kawase A, Funai K, Iizuka S, Yamashita T, Oiwa H, Sugimura H (2016). Two cases of lung metastasis originating from acquired cystic kidney disease-associated carcinoma. Int J Clin Exp Pathol..

[55] Wender R, Fontham ETH, Barrera E, Colditz GA, Church TR, Ettinger DS (2013). American cancer society lung cancer screening guidelines. CA Cancer J Clin.

[56] Cetin K, Ettinger DS, Hei Y-J, O’Malley CD (2011). Survival by histologic subtype in stage IV nonsmall cell lung cancer based on data from the surveillance, epidemiology and end results program. Clin Epidemiol.

[57] Kim ES (2016). Chemotherapy resistance in lung cancer. Adv Exp Med Biol.

[58] Chen MB, Whisler JA, Fröse J, Yu C, Shin Y, Kamm RD (2017). On-chip human microvasculature assay for visualization and quantification of tumor cell extravasation dynamics. Nat Protoc.

[59] Valastyan S, Weinberg RA (2011). Tumor metastasis: molecular insights and evolving paradigms. Cell.

[60] Portillo-Lara R, Annabi N (2016). Microengineered cancer-on-a-chip platforms to study the metastatic microenvironment. Lab Chip.

[61] Martin Tracey A YL, Sanders Andrew J, Lane Jane, Jiang Wen G. Cancer invasion and metastasis: molecular and cellular perspective. Madame Curie Bioscience Database. https://www.ncbi.nlm.nih.gov/books/NBK164700/. Landes Bioscience; 2013. Accessed 1 Mar 2019.

[62] Keshamouni V, Arenberg D, Kalemkerian G (2009). Lung cancer metastasis: novel biological mechanisms and impact on clinical practice.

[63] Alizadeh AM, Shiri S, Farsinejad S (2014). Metastasis review: from bench to bedside. Tumour Biol J Int Soc Oncodev Biol Med.

[64] Jiang WG, Sanders AJ, Katoh M, Ungefroren H, Gieseler F, Prince M (2015). Tissue invasion and metastasis: molecular, biological and clinical perspectives. Semin Cancer Biol.

[65] Syn N, Wang L, Sethi G, Thiery JP, Goh BC (2016). Exosome-mediated metastasis: from epithelial-mesenchymal transition to escape from immunosurveillance. Trends Pharmacol Sci.

[66] Tommelein J, Verset L, Boterberg T, Demetter P, Bracke M, De Wever O (2015). Cancer-associated fibroblasts connect metastasis-promoting communication in colorectal cancer. Front Oncol.

[67] Hou JM, Krebs M, Ward T, Sloane R, Priest L, Hughes A (2011). Circulating tumor cells as a window on metastasis biology in lung cancer. Am J Pathol.

[68] Popper HH (2016). Progression and metastasis of lung cancer. Cancer Metastasis Rev.

[69] Peinado H, Zhang H, Matei IR, Costa-Silva B, Hoshino A, Rodrigues G (2017). Pre-metastatic niches: organ-specific homes for metastases. Nat Rev Cancer.

[70] Nguyen DX, Bos PD, Massague J (2009). Metastasis: from dissemination to organ-specific colonization. Nat Rev Cancer.

[71] Nishida N, Yano H, Nishida T, Kamura T, Kojiro M (2006). Angiogenesis in Cancer. Vasc Health Risk Manag.

[72] Xu H, Li Z, Guo Y, Peng X, Qin J (2017). Probing the response of lung tumor cells to inflammatory microvascular endothelial cells on fluidic microdevice. Electrophoresis.

[73] Benoit MR, Conant CG, Ionescu-Zanetti C, Schwartz M, Matin A (2010). New device for high-throughput viability screening of flow biofilms. Appl Environ Microbiol.

[74] Guo T, Kong J, Liu Y, Li Z, Xia J, Zhang Y (2017). Transcriptional activation of NANOG by YBX1 promotes lung cancer stem-like properties and metastasis. Biochem Biophys Res Commun.

[75] Yu T, Guo Z, Fan H, Song J, Liu Y, Gao Z (2016). Cancer-associated fibroblasts promote non-small cell lung cancer cell invasion by upregulation of glucose-regulated protein 78 (GRP78) expression in an integrated bionic microfluidic device. Oncotarget..

[76] Cui X, Guo W, Sun Y, Sun B, Hu S, Sun D (2017). A microfluidic device for isolation and characterization of transendothelial migrating cancer cells. Biomicrofluidics.

[77] Kao Y-C, Hsieh M-H, Liu C-C, Pan H-J, Liao W-Y, Cheng J-Y (2014). Modulating chemotaxis of lung cancer cells by using electric fields in a microfluidic device. Biomicrofluidics.

[78] Zou H, Yue W, Yu WK, Liu D, Fong CC, Zhao J (2015). Microfluidic platform for studying chemotaxis of adhesive cells revealed a gradient-dependent migration and acceleration of cancer stem cells. Anal Chem.

[79] Li Y, Xu T, Zou H, Chen X, Sun D, Yang M (2017). Cell migration microfluidics for electrotaxis-based heterogeneity study of lung cancer cells. Biosens Bioelectron.

[80] Li Y, Xu T, Chen X, Lin S, Cho M, Sun D (2017). Effects of direct current electric fields on lung cancer cell electrotaxis in a PMMA-based microfluidic device. Anal Bioanal Chem.

[81] Fischer KR, Durrans A, Lee S, Sheng J, Li F, Wong S (2015). EMT is not required for lung metastasis but contributes to chemoresistance. Nature.

[82] Quail DF, Joyce JA (2013). Microenvironmental regulation of tumor progression and metastasis. Nat Med.

[83] Rivera A, Fu X, Tao L, Zhang X (2015). Expression of mouse CD47 on human cancer cells profoundly increases tumor metastasis in murine models. BMC Cancer..

[84] Bekes EM, Schweighofer B, Kupriyanova TA, Zajac E, Ardi VC, Quigley JP (2011). Tumor-recruited neutrophils and neutrophil TIMP-free MMP-9 regulate coordinately the levels of tumor angiogenesis and efficiency of malignant cell intravasation. Am J Pathol.

[85] Jeon JS, Zervantonakis IK, Chung S, Kamm RD, Charest JL (2013). In vitro model of tumor cell extravasation. PLoS ONE.

[86] Eckert MA, Lwin TM, Chang AT, Kim J, Danis E, Ohno-Machado L (2011). Twist1-induced invadopodia formation promotes tumor metastasis. Cancer Cell.

[87] D’Antonio C, Passaro A, Gori B, Del Signore E, Migliorino MR, Ricciardi S (2014). Bone and brain metastasis in lung cancer: recent advances in therapeutic strategies. Ther Adv Med Oncol.

[88] Lee H, Park W, Ryu H, Jeon NL (2014). A microfluidic platform for quantitative analysis of cancer angiogenesis and intravasation. Biomicrofluidics.

[89] Shin MK, Kim SK, Jung H (2011). Integration of intra- and extravasation in one cell-based microfluidic chip for the study of cancer metastasis. Lab Chip.

[90] Zhang Q, Liu T, Qin J (2012). A microfluidic-based device for study of transendothelial invasion of tumor aggregates in realtime. Lab Chip.

[91] Alonzo LF, Moya ML, Shirure VS, George SC (2015). Microfluidic device to control interstitial flow-mediated homotypic and heterotypic cellular communication. Lab Chip.

[92] Jusoh N, Oh S, Kim S, Kim J, Jeon NL (2015). Microfluidic vascularized bone tissue model with hydroxyapatite-incorporated extracellular matrix. Lab Chip.

[93] Kim J, Yang K, Park H-J, Cho S-W, Han S, Shin Y (2014). Implantable microfluidic device for the formation of three-dimensional vasculature by human endothelial progenitor cells. Biotechnol Bioprocess Eng.

[94] Wang X, Phan DT, Sobrino A, George SC, Hughes CC, Lee AP (2016). Engineering anastomosis between living capillary networks and endothelial cell-lined microfluidic channels. Lab Chip.

[95] Nassiri SM, Rahbarghazi R (2014). Interactions of mesenchymal stem cells with endothelial cells. Stem Cells Dev.

[96] Mihalache A, Rogoveanu I (2013). Angiogenesis factors involved in the pathogenesis of colorectal cancer. Curr Health Sci J..

[97] Klagsbrun M (1991). Regulators of angiogenesis: stimulators, inhibitors, and extracellular matrix. J Cell Biochem.

[98] Shi JUN, Wei P-K (2016). Interleukin-8: a potent promoter of angiogenesis in gastric cancer. Oncol Lett.

[99] Ning Y, Manegold PC, Hong YK (2011). Interleukin-8 is associated with proliferation, migration, angiogenesis and chemosensitivity in vitro and in vivo in colon cancer cell line models. Int J Cancer.

[100] Szabo V, Bugyik E, Dezso K, Ecker N, Nagy P, Timar J (2015). Mechanism of tumour vascularization in experimental lung metastases. J Pathol.

[101] Huang Y, Agrawal B, Sun D, Kuo JS, Williams JC (2011). Microfluidics-based devices: new tools for studying cancer and cancer stem cell migration. Biomicrofluidics.

[102] Kedrin D, van Rheenen J, Hernandez L, Condeelis J, Segall JE (2007). Cell motility and cytoskeletal regulation in invasion and metastasis. J Mammary Gland Biol Neoplasia.

[103] Mierke CT (2015). Physical view on migration modes. Cell Adhes Migrat.

[104] Wu J, Chen Q, Liu W, He Z, Lin J-M (2017). Recent advances in microfluidic 3D cellular scaffolds for drug assays. TrAC Trends Anal Chem.

[105] Li J, Lin F (2011). Microfluidic devices for studying chemotaxis and electrotaxis. Trends Cell Biol.

[106] Hou H-S, Tsai H-F, Chiu H-T, Cheng J-Y (2014). Simultaneous chemical and electrical stimulation on lung cancer cells using a multichannel-dual-electric-field chip. Biomicrofluidics.

[107] Hou HS, Chang HF, Cheng JY (2015). Electrotaxis studies of lung cancer cells using a multichannel dual-electric-field microfluidic chip. J Visual Exp JoVE..

[108] O’Flaherty JD, Gray S, Richard D, Fennell D, O’Leary JJ, Blackhall FH (2012). Circulating tumour cells, their role in metastasis and their clinical utility in lung cancer. Lung Cancer (Amsterdam, Netherlands)..

[109] Wang C, Ye M, Cheng L, Li R, Zhu W, Shi Z (2015). Simultaneous isolation and detection of circulating tumor cells with a microfluidic silicon-nanowire-array integrated with magnetic upconversion nanoprobes. Biomaterials.

[110] Wang J, Lu W, Tang C, Liu Y, Sun J, Mu X (2015). Label-free isolation and mRNA detection of circulating tumor cells from patients with metastatic lung cancer for disease diagnosis and monitoring therapeutic efficacy. Anal Chem.

[111] Poudineh M, Aldridge PM, Ahmed S, Green BJ, Kermanshah L, Nguyen V (2017). Tracking the dynamics of circulating tumour cell phenotypes using nanoparticle-mediated magnetic ranking. Nat Nano..

[112] Au SH, Edd J, Stoddard AE, Wong KHK, Fachin F, Maheswaran S (2017). Microfluidic isolation of circulating tumor cell clusters by size and asymmetry. Scientific Rep.

[113] Antfolk M, Kim SH, Koizumi S, Fujii T, Laurell T (2017). Label-free single-cell separation and imaging of cancer cells using an integrated microfluidic system. Sci Rep..

[114] Sarioglu AF, Aceto N, Kojic N, Donaldson MC, Zeinali M, Hamza B (2015). A microfluidic device for label-free, physical capture of circulating tumor cell clusters. Nat Meth..

[115] Song Y, Tian T, Shi Y, Liu W, Zou Y, Khajvand T (2017). Enrichment and single-cell analysis of circulating tumor cells. Chem Sci.

[116] Qian W, Zhang Y, Chen W (2015). Capturing cancer: emerging microfluidic technologies for the capture and characterization of circulating tumor cells. Small.

[117] Green BJ, Kermanshah L, Labib M, Ahmed SU, Silva PN, Mahmoudian L (2017). Isolation of phenotypically distinct cancer cells using nanoparticle-mediated sorting. ACS Appl Mater Interfaces.

[118] Jiang J, Zhao H, Shu W, Tian J, Huang Y, Song Y (2017). An integrated microfluidic device for rapid and high-sensitivity analysis of circulating tumor cells. Sci Rep..

[119] Raimondi C, Nicolazzo C, Gradilone A (2015). Circulating tumor cells isolation: the “post-EpCAM era”. Chin J Cancer Res.

[120] Lai CH, Choon Lim S, Wu LC, Wang CF, Tsai WS, Wu HC (2017). Site-specific antibody modification and immobilization on a microfluidic chip to promote the capture of circulating tumor cells and microemboli. Chem Commun (Cambridge)..

[121] Pu K, Li C, Zhang N, Wang H, Shen W, Zhu Y (2017). Epithelial cell adhesion molecule independent capture of non-small lung carcinoma cells with peptide modified microfluidic chip. Biosens Bioelectron.

[122] Bu J, Kim YJ, Kang YT, Lee TH, Kim J, Cho YH (2017). Polyester fabric sheet layers functionalized with graphene oxide for sensitive isolation of circulating tumor cells. Biomaterials.

[123] Hyun K-A, Lee TY, Jung H-I (2013). Negative enrichment of circulating tumor cells using a geometrically activated surface interaction chip. Anal Chem.

[124] Pedrol E, Garcia-Algar M, Massons J (2017). Optofluidic device for the quantification of circulating tumor cells in breast cancer. Sci Rep..

[125] Gleghorn JP, Pratt ED, Denning D, Liu H, Bander NH, Tagawa ST (2010). Capture of circulating tumor cells from whole blood of prostate cancer patients using geometrically enhanced differential immunocapture (GEDI) and a prostate-specific antibody. Lab Chip.

[126] Chikaishi Y, Yoneda K, Ohnaga T, Tanaka F (2017). EpCAM-independent capture of circulating tumor cells with a ‘universal CTC-chip’. Oncol Rep.

[127] Sheng W, Chen T, Kamath R, Xiong X, Tan W, Fan ZH (2012). Aptamer-enabled efficient isolation of cancer cells from whole blood using a microfluidic device. Anal Chem.

[128] Zhang J, Li S, Liu F, Zhou L, Shao N, Zhao X (2015). SELEX aptamer used as a probe to detect circulating tumor cells in peripheral blood of pancreatic cancer patients. PLoS ONE.

[129] Peng J, Zhao Q, Zheng W, Li W, Li P, Zhu L (2017). Peptide-functionalized nanomaterials for the efficient isolation of HER2-positive circulating tumor cells. ACS Appl Mater Interfaces.

[130] Poudineh M, Labib M, Ahmed S, Nguyen LN, Kermanshah L, Mohamadi RM (2017). Profiling functional and biochemical phenotypes of circulating tumor cells using a two-dimensional sorting device. Angew Chem Int Ed Engl.

[131] Jack R, Hussain K, Rodrigues D, Zeinali M, Azizi E, Wicha M (2017). Microfluidic continuum sorting of sub-populations of tumor cells via surface antibody expression levels. Lab Chip.

[132] Leggett SE, Wong IY (2017). Nanomedicine: catching tumour cells in the zone. Nat Nano..

[133] Bravo K, Ortega FG, Messina GA, Sanz MI, Fernández-Baldo MA, Raba J (2017). Integrated bio-affinity nano-platform into a microfluidic immunosensor based on monoclonal bispecific trifunctional antibodies for the electrochemical determination of epithelial cancer biomarker. Clin Chim Acta.

[134] Kwak B, Lee J, Lee D, Lee K, Kwon O, Kang S (2017). Selective isolation of magnetic nanoparticle-mediated heterogeneity subpopulation of circulating tumor cells using magnetic gradient based microfluidic system. Biosens Bioelectron.

[135] Xu H, Dong B, Xu S, Xu S, Sun X, Sun J (2017). High purity microfluidic sorting and in situ inactivation of circulating tumor cells based on multifunctional magnetic composites. Biomaterials.

[136] Sun N, Liu M, Wang J, Wang Z, Li X, Jiang B (2016). Chitosan nanofibers for specific capture and nondestructive release of CTCs assisted by pCBMA brushes. Small.

[137] Qiu J, Zhao K, Li L, Yu X, Guo W, Wang S (2017). A titanium dioxide nanorod array as a high-affinity nano-bio interface of a microfluidic device for efficient capture of circulating tumor cells. Nano Res.

[138] Park M-H, Reátegui E, Li W, Tessier SN, Wong KHK, Jensen AE (2017). Enhanced isolation and release of circulating tumor cells using nanoparticle binding and ligand exchange in a microfluidic chip. J Am Chem Soc.

[139] Shi G, Cui W, Benchimol M, Liu Y-T, Mattrey RF, Mukthavaram R (2013). Isolation of rare tumor cells from blood cells with buoyant immuno-microbubbles. PLoS ONE.

[140] Yang C, Zhang N, Wang S, Shi D, Zhang C, Liu K (2018). Wedge-shaped microfluidic chip for circulating tumor cells isolation and its clinical significance in gastric cancer. J Transl Med.

[141] Ren X, Foster BM, Ghassemi P, Strobl JS, Kerr BA, Agah M (2018). Entrapment of prostate cancer circulating tumor cells with a sequential size-based microfluidic chip. Anal Chem.

[142] Chiu T-K, Zhao Y, Chen D, Hsieh C-H, Wang K, Chou W-P (2017). A low-sample-loss microfluidic system for the quantification of size-independent cellular electrical property—its demonstration for the identification and characterization of circulating tumour cells (CTCs). Sens Actuators B Chem.

[143] Alshareef M, Metrakos N, Juarez Perez E, Azer F, Yang F, Yang X (2013). Separation of tumor cells with dielectrophoresis-based microfluidic chip. Biomicrofluidics.

[144] Chou W-P, Wang H-M, Chang J-H, Chiu T-K, Hsieh C-H, Liao C-J (2017). The utilization of optically-induced-dielectrophoresis (ODEP)-based virtual cell filters in a microfluidic system for continuous isolation and purification of circulating tumour cells (CTCs) based on their size characteristics. Sens Actuators B Chem.

[145] Che J, Sollier E, Go DE, Kummer N, Rettig M, Goldman J, Nickols N, McCloskey S, Kulkarni RP, Dino Di Carlo. Microfluidic vortex technology for pure circulating tumor cell concentration from patient blood. In: 17th International conference on miniaturized systems for chemistry and life sciences 2013.

[146] Chen H, Cao B, Sun B, Cao Y, Yang K, Lin Y-S (2017). Highly-sensitive capture of circulating tumor cells using micro-ellipse filters. Scientific Rep.

[147] Hosokawa M, Hayata T, Fukuda Y, Arakaki A, Yoshino T, Tanaka T (2010). Size-selective microcavity array for rapid and efficient detection of circulating tumor cells. Anal Chem.

[148] Warkiani ME, Khoo BL, Tan DS, Bhagat AA, Lim WT, Yap YS (2014). An ultra-high-throughput spiral microfluidic biochip for the enrichment of circulating tumor cells. Analyst..

[149] Kolostova K, Zhang Y, Hoffman RM, Bobek V (2014). In vitro culture and characterization of human lung cancer circulating tumor cells isolated by size exclusion from an orthotopic nude-mouse model expressing fluorescent protein. J Fluorescence.

[150] Kim TH, Lim M, Park J, Oh JM, Kim H, Jeong H (2017). FAST: size-selective, clog-free isolation of rare cancer cells from whole blood at a liquid-liquid interface. Anal Chem.

[151] Zhou M-D, Hao S, Williams AJ, Harouaka RA, Schrand B, Rawal S (2014). Separable bilayer microfiltration device for viable label-free enrichment of circulating tumour cells. Scientific Rep.

[152] Yang L, Tong X, Yucheng X, Dongyang K, Lei X, Jungwook P (2015). Isolation of circulating tumor cells by a magnesium-embedded filter. J Micromech Microeng.

[153] Kang HM, Kim GH, Jeon HK (2017). Circulating tumor cells detected by lab-on-a-disc: role in early diagnosis of gastric cancer. PLoS ONE.

[154] Gao W, Yuan H, Jing F (2017). Analysis of circulating tumor cells from lung cancer patients with multiple biomarkers using high-performance size-based microfluidic chip. Oncotarget..

[155] Jensen KH, Valente AX, Stone HA (2014). Flow rate through microfilters: influence of the pore size distribution, hydrodynamic interactions, wall slip, and inertia. Phys Fluids.

[156] Zhang Z, Xu J, Hong B, Chen X (2014). The effects of 3D channel geometry on CTC passing pressure–towards deformability-based cancer cell separation. Lab Chip.

[157] Regmi S, Fu A, Luo KQ (2017). High shear stresses under exercise condition destroy circulating tumor cells in a microfluidic system. Scientific Rep.

[158] Khoo BL, Warkiani ME, Tan DS-W, Bhagat AAS, Irwin D, Lau DP (2014). Clinical validation of an ultra high-throughput spiral microfluidics for the detection and enrichment of viable circulating tumor cells. PLoS ONE..

[159] Kulasinghe A, Tran THP, Blick T, O’Byrne K, Thompson EW, Warkiani ME (2017). Enrichment of circulating head and neck tumour cells using spiral microfluidic technology. Scientific Rep.

[160] Kim B, Lee JK, Choi S (2016). Continuous sorting and washing of cancer cells from blood cells by hydrophoresis. BioChip J.

[161] Hyun K-A, Koo G-B, Han H, Sohn J, Choi W, Kim S-I (2016). Epithelial-to-mesenchymal transition leads to loss of EpCAM and different physical properties in circulating tumor cells from metastatic breast cancer. Oncotarget..

[162] Khodaee F, Movahed S, Fatouraee N, Daneshmand F (2015). Numerical simulation of separation of circulating tumor cells from blood stream in deterministic lateral displacement (DLD) microfluidic channel. J Mech.

[163] Khojah R, Stoutamore R, Di Carlo D (2017). Size-tunable microvortex capture of rare cells. Lab Chip.

[164] Antfolk M, Antfolk C, Lilja H, Laurell T, Augustsson P (2015). A single inlet two-stage acoustophoresis chip enabling tumor cell enrichment from white blood cells. Lab Chip.

[165] Antfolk M, Magnusson C, Augustsson P, Lilja H, Laurell T (2015). Acoustofluidic, label-free separation and simultaneous concentration of rare tumor cells from white blood cells. Anal Chem.

[166] Li P, Mao Z, Peng Z, Zhou L, Chen Y, Huang P-H (2015). Acoustic separation of circulating tumor cells. Proc Natl Acad Sci USA.

[167] Moon HS, Kwon K, Kim SI, Han H, Sohn J, Lee S (2011). Continuous separation of breast cancer cells from blood samples using multi-orifice flow fractionation (MOFF) and dielectrophoresis (DEP). Lab Chip.

[168] Li M, Anand RK (2017). High-throughput selective capture of single circulating tumor cells by dielectrophoresis at a wireless electrode array. J Am Chem Soc.

[169] Lin YH, Yang YW, Chen YD, Wang SS, Chang YH, Wu MH (2012). The application of an optically switched dielectrophoretic (ODEP) force for the manipulation and assembly of cell-encapsulating alginate microbeads in a microfluidic perfusion cell culture system for bottom-up tissue engineering. Lab Chip.

[170] Chiu T-K, Chou W-P, Huang S-B, Wang H-M, Lin Y-C, Hsieh C-H (2016). Application of optically-induced-dielectrophoresis in microfluidic system for purification of circulating tumour cells for gene expression analysis-cancer cell line model. Scientific Rep.

[171] Alazzam A, Mathew B, Alhammadi F (2017). Novel microfluidic device for the continuous separation of cancer cells using dielectrophoresis. J Sep Sci.

[172] Fu Y, Yuan Q, Guo J (2017). Lab-on-PCB-based micro-cytometer for circulating tumor cells detection and enumeration. Microfluid Nanofluid.

[173] Chen W, Allen SG, Reka AK, Qian W, Han S, Zhao J (2016). Nanoroughened adhesion-based capture of circulating tumor cells with heterogeneous expression and metastatic characteristics. BMC Cancer..

[174] Kalinich M, Bhan I, Kwan TT (2017). An RNA-based signature enables high specificity detection of circulating tumor cells in hepatocellular carcinoma. Proc Natl Acad Sci USA..

[175] Che J, Yu V, Dhar M (2016). Classification of large circulating tumor cells isolated with ultra-high throughput microfluidic Vortex technology. Oncotarget..

[176] Sequist LV, Nagrath S, Toner M, Haber DA, Lynch TJ (2009). The CTC-chip: an exciting new tool to detect circulating tumor cells in lung cancer patients. J Thorac Oncol..

[177] Zhang Z, Shiratsuchi H, Lin J, Chen G, Reddy RM, Azizi E (2014). Expansion of CTCs from early stage lung cancer patients using a microfluidic co-culture model. Oncotarget..

[178] Che J, Yu V, Garon EB, Goldman JW, Di Carlo D (2017). Biophysical isolation and identification of circulating tumor cells. Lab Chip.

[179] Qian C, Wu S, Chen H, Zhang X, Jing R, Shen L (2018). Clinical significance of circulating tumor cells from lung cancer patients using microfluidic chip. Clin Exp Med.

[180] Dhar M, Wong J, Che J, Matsumoto M, Grogan T, Elashoff D (2018). Evaluation of PD-L1 expression on vortex-isolated circulating tumor cells in metastatic lung cancer. Scientific Rep.

[181] Doryab A, Amoabediny G, Salehi-Najafabadi A (2016). Advances in pulmonary therapy and drug development: lung tissue engineering to lung-on-a-chip. Biotechnol Adv.

[182] Punde TH, Wu W-H, Lien P-C, Chang Y-L, Kuo P-H, Chang MD-T (2015). A biologically inspired lung-on-a-chip device for the study of protein-induced lung inflammation. Integr Biol..

[183] Konar D, Devarasetty M, Yildiz DV, Atala A, Murphy SV (2016). Lung-on-A-chip technologies for disease modeling and drug development. Biomed Eng Comput Biol.

[184] Zhang YS, Zhang Y-N, Zhang W (2017). Cancer-on-a-chip systems at the frontier of nanomedicine. Drug Discov Today.

[185] Pradhan S, Smith AM, Garson CJ, Hassani I, Pant K, Arnold RD (2016). Microfluidic cancer-on-a-chip platform for assessing anti-cancer drug efficacies. Can Res.

[186] Astolfi M, Peant B, Lateef MA, Rousset N, Kendall-Dupont J, Carmona E (2016). Micro-dissected tumor tissues on chip: an ex vivo method for drug testing and personalized therapy. Lab Chip.

[187] Skardal A, Devarasetty M, Forsythe S, Atala A, Soker S (2016). A reductionist metastasis-on-a-chip platform for in vitro tumor progression modeling and drug screening. Biotechnol Bioeng.

[188] Huh D, Matthews BD, Mammoto A, Montoya-Zavala M, Hsin HY, Ingber DE (2010). Reconstituting organ-level lung functions on a chip. Science.

[189] Xu Z, Li E, Guo Z, Yu R, Hao H, Xu Y (2016). Design and construction of a multi-organ microfluidic chip mimicking the in vivo microenvironment of lung cancer metastasis. ACS Appl Mater Interfaces.

[190] Sobrino A, Phan DTT, Datta R, Wang X, Hachey SJ, Romero-López M (2016). 3D microtumors in vitro supported by perfused vascular networks. Scientific Rep.

[191] Kapałczyńska M, Kolenda T, Przybyła W (2016). 2D and 3D cell cultures—a comparison of different types of cancer cell cultures. Arch Med Sci..

[192] Ronaldson-Bouchard K, Vunjak-Novakovic G (2018). Organs-on-a-chip: a fast track for engineered human tissues in drug development. Cell Stem Cell.

[193] Biselli E, Agliari E, Barra A, Bertani FR, Gerardino A, De Ninno A (2017). Organs on chip approach: a tool to evaluate cancer -immune cells interactions. Scientific Rep.

[194] Onion D, Argent RH, Reece-Smith AM, Craze ML, Pineda RG, Clarke PA (2016). 3-Dimensional patient-derived lung cancer assays reveal resistance to standards-of-care promoted by stromal cells but sensitivity to histone deacetylase inhibitors. Mol Cancer Ther.

[195] Chidambaram M, Manavalan R, Kathiresan K (2011). Nanotherapeutics to overcome conventional cancer chemotherapy limitations. J Pharm Pharm Sci.

[196] Funkhouser J (2002). Reinventing pharma: the theranostic revolution. Curr Drug Discov.

[197] Luk BT, Zhang L (2014). Current advances in polymer-based nanotheranostics for cancer treatment and diagnosis. ACS Appl Mater Interfaces.

[198] Wang Z, Qiao R, Tang N, Lu Z, Wang H, Zhang Z (2017). Active targeting theranostic iron oxide nanoparticles for MRI and magnetic resonance-guided focused ultrasound ablation of lung cancer. Biomaterials.

[199] Howell M, Mallela J, Wang C (2013). Manganese-loaded lipid-micellar theranostics for simultaneous drug and gene delivery to lungs. J Control Release.

[200] Muthu MS, Feng SS (2013). Theranostic liposomes for cancer diagnosis and treatment: current development and pre-clinical success. Expert Opin Drug Deliv.

[201] Wu YF, Wu HC, Kuan CH (2016). Multi-functionalized carbon dots as theranostic nanoagent for gene delivery in lung cancer therapy. Sci Rep..

[202] Han G, Chen G (2013). Theranostic upconversion nanoparticles (II). Theranostics..

[203] Du G, Fang Q, den Toonder JMJ (2016). Microfluidics for cell-based high throughput screening platforms—a review. Anal Chim Acta.

[204] Zhao L, Wang Z, Fan S, Meng Q, Li B, Shao S (2010). Chemotherapy resistance research of lung cancer based on micro-fluidic chip system with flow medium. Biomed Microdevice.

[205] Gao D, Liu J, Wei H-B, Li H-F, Guo G-S, Lin J-M (2010). A microfluidic approach for anticancer drug analysis based on hydrogel encapsulated tumor cells. Anal Chim Acta.

[206] Xu Z, Gao Y, Hao Y, Li E, Wang Y, Zhang J (2013). Application of a microfluidic chip-based 3D co-culture to test drug sensitivity for individualized treatment of lung cancer. Biomaterials.

[207] Dereli-Korkut Z, Akaydin HD, Ahmed AH, Jiang X, Wang S (2014). Three dimensional microfluidic cell arrays for ex vivo drug screening with mimicked vascular flow. Anal Chem.

[208] Ying L, Zhu Z, Xu Z, He T, Li E, Guo Z (2015). Cancer associated fibroblast-derived hepatocyte growth factor inhibits the paclitaxel-induced apoptosis of lung cancer A549 cells by up-regulating the PI3K/Akt and GRP78 signaling on a microfluidic platform. PLoS ONE.

[209] Teeguarden JG, Hinderliter PM, Orr G, Thrall BD, Pounds JG (2007). Particokinetics in vitro: dosimetry considerations for in vitro nanoparticle toxicity assessments. Toxicol Sci.

[210] Park MS, Yoon TH (2014). Effects of Ag nanoparticle flow rates on the progress of the cell cycle under continuously flowing “dynamic” exposure conditions. B Korean Chem Soc..

[211] Mahto SK, Yoon TH, Rhee SW (2010). A new perspective on in vitro assessment method for evaluating quantum dot toxicity by using microfluidics technology. Biomicrofluidics.

[212] Richter L, Charwat V, Jungreuthmayer C, Bellutti F, Brueckl H, Ertl P (2011). Monitoring cellular stress responses to nanoparticles using a lab-on-a-chip. Lab Chip.

[213] Mahto SK, Yoon TH, Rhee SW (2010). Cytotoxic effects of surface-modified quantum dots on neuron-like PC12 cells cultured inside microfluidic devices. BioChip J.

[214] Li YS, Haga JH, Chien S (2005). Molecular basis of the effects of shear stress on vascular endothelial cells. J Biomech.

[215] Kim D, Lin YS, Haynes CL (2011). On-chip evaluation of shear stress effect on cytotoxicity of mesoporous silica nanoparticles. Anal Chem.

[216] Samuel SP, Jain N, O’Dowd F (2012). Multifactorial determinants that govern nanoparticle uptake by human endothelial cells under flow. Int J Nanomed.

[217] Fede C, Albertin G, Petrelli L, De Caro R, Fortunati I, Weber V (2017). Influence of shear stress and size on viability of endothelial cells exposed to gold nanoparticles. J Nanopart Res.

[218] Singh R, Lillard JW (2009). Nanoparticle-based targeted drug delivery. Exp Mol Pathol.

[219] Korin N, Kanapathipillai M, Matthews BD, Crescente M (2012). Shear-activated nanotherapeutics for drug targeting to obstructed blood vessels. Science.

[220] Holme MN, Fedotenko IA, Abegg D, Althaus J, Babel L, Favarger F (2012). Shear-stress sensitive lenticular vesicles for targeted drug delivery. Nat Nanotechnol.

[221] Paulis LE, Jacobs I, van den Akker NM, Geelen T, Molin DG, Starmans LW (2012). Targeting of ICAM-1 on vascular endothelium under static and shear stress conditions using a liposomal Gd-based MRI contrast agent. J Nanobiotechnol.

[222] Hosta-Rigau L, Stadler B (2013). Shear stress and its effect on the interaction of myoblast cells with nanosized drug delivery vehicles. Mol Pharm.

[223] Teo BM, van der Westen R, Hosta-Rigau L, Stadler B (2013). Cell response to PEGylated poly(dopamine) coated liposomes considering shear stress. Bba-Gen Subjects..

[224] Kim Y, Lobatto ME, Kawahara T, Lee Chung B, Mieszawska AJ, Sanchez-Gaytan BL (2014). Probing nanoparticle translocation across the permeable endothelium in experimental atherosclerosis. Proc Natl Acad Sci USA.

[225] Albanese A, Lam AK, Sykes EA, Rocheleau JV, Chan WC (2013). Tumour-on-a-chip provides an optical window into nanoparticle tissue transport. Nat Commun..

[226] Li X, Ballerini DR, Shen W (2012). A perspective on paper-based microfluidics: current status and future trends. Biomicrofluidics.

[227] Lisowski P, Zarzycki PK (2013). Microfluidic paper-based analytical devices (μPADs) and micro total analysis systems (μTAS): development, applications and future trends. Chromatographia.

[228] El-Ali J, Sorger PK, Jensen KF (2006). Cells on chips. Nature.

[229] Long C, Finch C, Esch M, Anderson W, Shuler M, Hickman J (2012). Design optimization of liquid-phase flow patterns for microfabricated lung on a chip. Ann Biomed Eng.

[230] Palaninathan V, Kumar V, Maekawa T, Liepmann D, Paulmurugan R, Eswara JR (2018). Multi-organ on a chip for personalized precision medicine. MRS Commun.

[231] Pham QN, Trinh KTL, Jung SW, Lee NY (2018). Microdevice-based solid-phase polymerase chain reaction for rapid detection of pathogenic microorganisms. Biotechnol Bioeng.

[232] Tharakan R, Tao D, Ubaida-Mohien C, Dinglasan RR, Graham DR (2015). Integrated microfluidic chip and online SCX separation allows untargeted nanoscale metabolomic and peptidomic profiling. J Proteome Res.

[233] Peng W, Unutmaz D, Ozbolat IT (2016). Bioprinting towards physiologically relevant tissue models for pharmaceutics. Trends Biotechnol.

